# 

**Published:** 1897-12

**Authors:** 


### The Library of the Bristol Medico-Chirurgical Society.

At the Annual Meeting, held on October 13th, 1897, Mr. L. M. Griffiths, the Honorary Librarian, read the following Report:—

The Library of the Bristol Medico-Chirurgical Society now consists, including 574 duplicates, of 5,833 volumes. 872 volumes have been added during the year, and 162 have been given to other Libraries or sold. We are in regular receipt of 172 periodicals.

Up to September 30th the donors of books since the last report have been: The American Public Health Association, Dr. J. Michell Clarke, the Columbian University, Dr. Carey Coombs, the Detroit Public Library, Mr. J. Ewens, Dr. G. M. Gould, Mr. L. M. Griffiths, Mr. W. H. Harsant, Mr. C. B. Keetley, the Laryngological Society of London, the Pathological Society of London, Mr. Young J. Pentland, Lady Reynolds, the Royal Academy of Medicine in Ireland, the Royal Medical and Chirurgical Society, Mr. J. Greig Smith, Mrs. J. Greig Smith, Dr. R. Shingleton Smith, the Smithsonian Institution, the Surgeon-General United States Army, and Messrs. J. Wright & Co. Framed pictures have been presented by Mr. L. M. Griffiths, Mr. Arthur W. Prichard, Dr. E. Markham Skerritt, and Mrs. J. Greig Smith.

The Library Committee has sustained a severe loss by the death of its Chairman, Mr. Greig Smith. The June number of our *Journal* paid a tribute to his worth in this capacity, but I may be permitted to supplement that by saying that the library could not have had a more single-hearted champion than Greig Smith, who was ever ready to devise schemes to promote its welfare and to insist that local professional life could not thrive without it.

Considering the number of new members elected during each session of the Society, it is well to state from time to time that the Bristol Medical Library, to which members of this Society have access, consists of the Library of the Faculty of Medicine of Bristol University College combined with that of our Society, and that in addition the Libraries of the Infirmary and Hospital are here on permanent loan. This arrangement gives a collection at present of 16,662 volumes, with a list of 206 current periodicals.

The duplicates now form by themselves a large and useful library, and under certain easy conditions they can be taken out on loan.

Much progress has been made with the cataloguing of the whole library, and there is every reason to believe that the increasing number of readers find the Card Catalogue indispensable in their research. The Clerk and Assistant-Clerk show great intelligence, not only in respect to the work of cataloguing, but also in the general assistance they are able to render all students and readers in the library.

Many of the important sets of periodicals, including *Hospital Reports* and *Society Transactions*, are now complete, and many others are very nearly so. But in foreign periodicals we need much to make up our files, and the Library Committee will be grateful for any volumes or numbers useful for this purpose, as the purchase of these would quickly absorb all available funds. We shall also be glad to have books of any kind, even if their literary value is not apparent at first sight.

Since this time last year a great improvement has been effected in the library by the substitution of incandescent burners for the ordinary gas jets; and in the provision of other appliances the College authorities have shown a careful regard for facilitating all library work.

I have no wish to hasten the removal of any particular doctor to another world, but as it is a common thing for large institutions to provide their friends and supporters with a form of bequest, I may be allowed to go so far as to suggest to every practitioner in the district, in case his will does not already contain an unequivocal clause bequeathing his medical books to this library, the desirability of adding without delay a codicil to that effect. This will ensure a due and thoughtful care of the books to which he was so fondly attached, and perhaps spare his soul the vexation which might arise from the knowledge that they had fallen into unloving and unappreciative hands.

*The following donations have been received since the publication of the list in September:*

*November 30th, 1897.*

| | |
|---|---|
| W. F. Cleveland, M.D. (1) ... ... ... ... ... | 1 volume. |
| Detroit Public Library (2) ... ... ... ... ... | 1 ,, |
| Dundee Medical Library (3) ... ... .. ... ... | 4 volumes. |
| L. M. Griffiths (4) ... ... ... ... ... ... ... | 2 ,, |
| Orleans Parish Medical Society (5) ... ... ... | 11 ,, |
| Mrs. Greig Smith (6) ... ... ... ... ... ... | 538 ,, |
| R. Shingleton Smith, M.D. (7) ... ... ... ... | 6 ,, |

Reprints have been received from Mr. L. M. Griffiths and Mrs. Greig Smith; unbound periodicals from Dundee Medical Library, Orleans Parish Medical Society and Mrs. Greig Smith. Five framed pictures have been presented by Mrs. Greig Smith, one by Dr. A. J. Harrison, *Asclepiad* portraits by Mr. Bertram Richardson, and some unframed portraits by Dr. J. O. Symes.

### TWENTY-SIXTH LIST OF BOOKS.

The titles of books mentioned in previous lists are not repeated.

The figures in brackets refer to the figures after the names of the donors, and show by whom the volumes were presented. The books to which no such figures are attached have either been bought from the Library Fund or received through the *Journal.*

| | | | |
|---|---|---|---|
| **Adams, W.** ... ... | *Club-Foot* ... ... ... ... ... ... ... ... ... | (6) | 1866 |
| **Aitchison, R. S.** ... | *Synopsis of Therapeutics* ... ... ... ... ... ... | (6) | 1886 |
| **Aitken, Sir W.** ... | *The Growth of the Recruit* ... ... ... | (6) 2nd Ed. | 1887 |
| ,, ... | *Animal Alkaloids* ... ... ... ... ... | (6) 2nd Ed. | 1889 |
| **Aldersmith, H.** ... | *Ringworm and Alopecia Areata* ... ... ... | 4th Ed. | 1897 |
| **Alexander, W.** ... | *Shortening the Round Ligaments* ... ... ... ... | (6) | 1884 |
| ,, ... | *The Treatment of Epilepsy* ... ... ... ... ... | (6) | 1889 |
| **Allbutt, T. C.** [Ed.] | *A System of Medicine.* Vol. IV.... ... ... ... ... ... | | 1897 |
| **Allbutt and T. P. Teale, T.C.** | *Clinical Lectures* ... ... ... ... ... | (6) | 1885 |
| **Allingham, H. W.** | *Colotomy* ... ... ... ... ... ... ... ... ... ... | (6) | 1892 |
| **Althaus, J.** ... ... | *Syphilis of the Nervous System* ... ... ... ... | (6) | 1890 |
| **Anderson, A.** ... | *Study of Fever* ... ... ... ... ... ... ... ... ... | (6) | 1861 |
| **Andral, G.** ... ... | *Pathological Anatomy.* (Tr. by R. Townsend and W. West) 2 vols. ... ... ... ... | (6) | 1829-31 |
| **Aveling, J. H.** ... | *On Inversion of the Uterus* ... ... ... ... ... | (6) | 1886 |
| **Baber, E. C.** ... ... | *A Guide to the Examination of the Nose* ... ... | (6) | 1886 |
| **Baily, A.** ... ... | *De Hydrope Anasarca* ... ... ... ... ... ... | (6) | 1723 |
| **Bain, A.** ... ... | *On the Study of Character* ... ... ... ... ... | (6) | 1861 |
| **Balfour, G. W.** ... | *Diseases of the Heart and Aorta* ... ... ... ... | (6) | 1876 |
| **Banks, W. M.** ... | *Clinical Notes upon Two Years' Surgical Work in the Liverpool Royal Infirmary* ... ... ... ... | (6) | 1884 |
| **Bantock, G. G.** ... | *Early Ovariotomy* ... ... ... ... ... ... ... ... | (6) | 1881 |
| ,, ... | *Rupture of the Female Perineum* ... ... | (6) 2nd Ed. | 1888 |

**Barrett, A. W.** ... *Dental Surgery* ... (6) 1885
**Barwell, R.** ... *On Aneurism* ... (6) 1880
,, ... *Diseases of the Joints* ... (6) 2nd Ed. 1881
**Bastian, H. C.** ... *Paralyses: Cerebral, Bulbar and Spinal* ... (6) 1886
*Bath as a Health Resort* ... [N.D.]
**Beaney, J. G.** ... *Constitutional Syphilis* ... (6) 3rd Ed. 1878
**Begbie, J. W.** ... *The Causes of Death among the Assured of the Scottish Widows' Fund Life Assurance Society*, 1867–73 (6) 1874
**Bennet, J. H.** ... *Inflammation of the Uterus* ... (6) 2nd Ed. 1849
**Benton, S.** ... *Diseases of the Rectum* ... (6) 1886
**Berkart, J. B.** ... *On Bronchial Asthma* ... (6) 2nd Ed. 1889
**Bigelow, H. R.** ... *Gynæcological Electro-Therapeutics* ... (6) 1889
**Bishop, E. S.** ... *Antiseptics in Surgery* ... (6) [N.D.]
**Bottentuit, [—]** ... *The Waters of Plombières (Vosges)* ... (6) 1888
**Bramwell, B.** ... *Pleurisy and Empyema* ... (6) 1889
**Brass, A.** ... *Atlas of Human Histology*. (Tr. by R. A. Young) ... 1897
**Braune, W.** ... *An Atlas of Topographical Anatomy*. (Tr. by E. Bellamy) ... (6) 1877
*British Physicians, Lives of* ... (6) 1830
**Broadbent, Sir W. H.** *The Pulse* ... (6) 1890
,, ,, **and J. F. H. Broadbent.** *Heart Disease* ... 1897
**Broca and F. Lubet-Barbon, A.** *Mastoid Abscesses and their Treatment*. (Tr. and Ed. by H. J. Curtis) ... 1897
**Brodie, Sir B. C.** ... *Psychological Inquiries* ... (6) 3rd Ed. 1856
,, ... *Works*. (Ed. by C. Hawkins) 3 vols. ... (6) 1865
**Brown, A. S.** ... *Madeira and the Canary Islands* ... (6) 2nd Ed. 1890
**Brown, H.** ... *Economics, Anæsthetics, and Antiseptics in the Practice of Midwifery* ... 1897
**Brown, J. J. G.** ... *Medical Diagnosis* ... 4th Ed. 1897
**Brown, T.** ... *Inquiry into the Relation of Cause and Effect* (6) 4th Ed. 1835
**Browne, L.** ... *The Throat and its Diseases* ... (6) 2nd Ed. 1887
**Browne, Sir T.** ... *Religio Medici* ... (6) 1886
**Buck, G.** ... *Reparative Surgery* ... (6) 1876
**Buist, J. B.** ... *Vaccinia and Variola* ... (6) 1887
**Burdett, H. C.** ... *The Relative Mortality of Large and Small Hospitals* (6) 1882
**C., I.** ... *Wits Interpreter, the English Parnassus* ... (6) 1655
**Campbell, H.** ... *The Causation of Disease* ... (6) 1889
,, ... *Flushing and Morbid Blushing* ... (6) 1890
**Celsus, A. C.** ... *De Medicina Libri Octo ex recensione L. Targae* ... (6) 1814
**Chaffey, W. C.** ... *Lymph-Stasis* ... (6) 1890
**Champneys, F. H.** *Artificial Respiration* ... (6) 1887
**Cheselden, W.** ... *On the High Operation for the Stone* ... (6) 1723
**Churchill, F.** ... *Face and Foot Deformities* ... (6) 1885
**Clarke, W. B.** ... *Surgery of the Kidney* ... (6) 1886
**Cleland and J. Y. Mackay, J.** *Human Anatomy* ... 1896
**Cleveland, W. F.** *On Induction* ... (1) 1897
**Clevenger, S. V.** ... *Spinal Concussion* ... (6) 1889
**Coats, W. T. Gairdner and J.** *Lectures to Practitioners* ... (6) 1888
**Cornil, V.** ... *Syphilis*. (Tr. by J. H. C. Simes and J. W. White) (6) 1882
**Cotterell, E.** ... *Injuries to Limbs* ... (6) 1885

**Courty, A.** ... *Diseases of the Uterus.* (Tr. from 3rd Ed. by Agnes M'Laren) ... (6) 1882
**Creighton, C.** ... *Unconscious Memory in Disease* ... (6) 1886
,, ... *Cow-Pox and Vaccinal Syphilis* ... (6) 1887
**Crooke, H.** ... *A Description of the Body of Man* ... (6) 1615
**Crookshank, E. M.** *Photography of Bacteria* ... (6) 1887
,, ... *History and Pathology of Vaccination.* 2 vols ... (6) 1889
**Curtis, H. R. Wharton and B. F.** *The Practice of Surgery* ... 1898
**Dalby, Sir W. B.** ... *Aural Surgery* ... (6) 1887
**Davey, J. G.** ... *The Ganglionic Nervous System* ... (6) 1858
**Davies, H.** ... *The Circulation through Diseased Hearts.* (Ed. by A. T. Davies) ... (6) 1889
**Day, W. H.** ... *On Irritable Brain in Children* ... (6) 1886
**Deaver, J. B.** ... *Appendicitis* ... (6) 1896
**Dekkers, F.** ... *Exercitiones Practicæ circa Medendi Methodum.* (6) Editio Altera 1695
**Demarquay, J. N.** *Medical Pneumatology.* (Tr. by S. S. Wallian) ... (6) 1889
**Distin-Maddick, E.** *Stricture of the Urethra* ... (6) 1887
**Draper, J. W.** ... *The Conflict between Religion and Science* (6) 8th Ed. 1876
**Duboué, H.** ... *Pau and its Neighbourhood.* (Tr. by R. de M. Clay) (6) 1882
**Dührssen, A.** ... *A Manual of Obstetric Practice.* (Tr. and Ed. from 6th Ed. by J. W. Taylor and F. Edge) ... 1897
**Duncan, A.** ... *Prevention of Disease in Tropical Campaigns* ... (6) 1888
**Dupouy, E.** ... *Medicine in the Middle Ages.* (Tr. by T. C. Minor) (6) 1889
**Eccles, A. S.** ... *The Practice of Massage* ... 2nd Ed. 1898
**Edis, A. W.** ... *Sterility in Women* ... (6) 1890
**Farquharson, R.** ... *A Guide to Therapeutics* (6) 3rd Ed., 1883; (6) 4th Ed. 1886
**Felkin, R. W.** ... *Geographical Distribution of Tropical Diseases* ... (6) 1889
**Fenelon, De la Motte** *Lives of the Ancient Philosophers* ... (6) [N.D.]
**Fenwick, E. H.** ... *The Electric Illumination of the Bladder and Urethra* (6) 1888
**Fenwick, S.** ... *The Saliva as a Test for Functional Disorders of the Liver* ... (6) 1887
,, **and W. S. Fenwick.** *Medical Diagnosis* ... 8th Ed. 1897
**Finlayson, J.** [Ed.] *Clinical Manual* ... (6) 2nd Ed. 1886
**Finny, J. M.** ... *Physical Diagnosis of Lung Diseases* ... (6) 1887
**Fothergill, J. M.** ... *Vaso-Renal Change* versus *Bright's Disease* ... (6) 1887
,, ... *Indigestion and Biliousness* ... (6) 2nd Ed. 1887
,, ... *The Practitioner's Handbook of Treatment.* 4th Ed. (Ed. by W. Murrell) ... 1897
**Fowler, G. R.** ... *Appendicitis* ... (6) 1894
**Fowler, J. K.** ... *The Localisation of the Lesions of Phthisis* ... (6) 1888
**Fox, E. L.** ... *The Influence of the Sympathetic on Disease* ... (6) 1885
**Foy, G.** ... *Anæsthetics* ... (6) 1889
**Freyer, P. J.** ... *Litholapaxy* ... (6) 1886
**Gairdner and J. Coats, W. T.** *Lectures to Practitioners* ... (6) 1888
**Geisse, N.** ... *A Guide to Ems* ... (6) 5th Ed 1883
*German Clinical Lectures* ... (6) 1876
**Gibney, V. P.** ... *The Hip and its Diseases* ... (6) 1884
**Glaister, J.** ... *Hygiene for Students and Nurses* ... 1897
*Gold-Headed Cane, The* ... (6) 1827

**Goodell, W.** ... *Lessons in Gynecology* ... (6) 3rd Ed. 1887
**Gordon, C. A.** ... *Rabies and Hydrophobia* ... (6) 1887
,, ... *Comments on the Report of the Committee on M. Pasteur's Treatment of Rabies and Hydrophobia* ... (6) 1888
**Gould, A. P.** ... *Elements of Surgical Diagnosis* ... (6) 1884
**Gowers, W. R.** ... *Diseases of the Spinal Cord* ... (6) 1880
,, ... *Diseases of the Brain* ... (6) 1885
**Gray, H.** ... *Anatomy* ... (6) 7th Ed. 1875
**Green, T. H.** ... *Pathology and Morbid Anatomy* ... (6) 3rd Ed. 1875
**Gresswell, J. B.** ... *Veterinary Pharmacology and Therapeutics* ... (6) 1885
**Gron, K.** ... *Studier over Gummos ("Tertiær") Syfilis* ... 1897
**Gross, S. W.** ... *Tumors of the Mammary Gland* ... (6) 1880
**Grün and W. D. Severn, E. F.** *Handbook to Dr. Koch's Treatment in Tubercular Disease* ... (6) [1890]
**Habershon, S. O.** ... *Diseases of the Abdomen* ... (6) 3rd Ed. 1878
**Hall, N.** ... *The Christian Philosopher* ... (6) [N.D.]
**Hamilton, D. J.** ... *A Text-Book of Pathology.* Vol. I. ... (6) 1889
**Harding, W.** ... *Fever Nursing* ... 1897
**Hare, H. A.** ... *Practical Diagnosis* ... 2nd Ed. 1897
,, ... *Text-Book of Practical Therapeutics* ... 6th Ed. 1897
**Harley, G.** ... *Inflammations of the Liver* ... (6) 1886
**Harrison, R.** ... *Lithotomy, Lithotrity, &c.* ... (6) 1883
,, ... *Surgical Disorders of the Urinary Organs* (6) 3rd Ed. 1887
,, ... *Urinary Disorders* ... (6) 1890
**Harvey, A.** ... *On the Fœtus in Utero* ... (6) 1886
**Harvey, W.** ... *Circulation of the Blood.* (Willis's Translation, Ed. by A. Bowie) ... (6) 1889
**Hawkins, H. P.** ... *Diseases of the Vermiform Appendix* ... (6) 1895
**Heanley, K. M.** ... *A Manual of Urine Testing* ... (6) 1886
**Heath, C.** ... *Injuries and Diseases of the Jaws* ... (6) 2nd Ed. 1872
**Hetling, W.** ... *Case of Osteo-Sarcoma of both Jaws* ... (6) [N.D.]
**Hill, M. B.** ... *Chronic Urethritis* ... (6) 1890
**Hillier, T.** ... *Hand-Book of Skin Diseases* ... (6) 1865
**Holmes, T.** ... *Surgery* (6) 1875 ; (6) 5th Ed. (Ed. by T. P. Pick) 1888
**Hood, D. W. C.** ... *Diseases and their Commencement* ... (6) 1886
**Humphrey, L.** ... *A Manual of Nursing* ... (6) 1889
**Hutchison and H. Rainy, R.** *Clinical Methods* ... 1897
**Huxley, T. H.** ... *Critiques and Addresses* ... (6) 1873
**Illingworth, C. R.** *The Abortive Treatment of Specific Febrile Disorders by the Biniodide of Mercury* ... (6) 1888
**James, B. W.** ... *American Resorts and Climates* ... (6) 1889
**James, P.** ... *Sore Throat* ... (6) 5th Ed. 1886
,, ... *Laryngoscopy and Rhinoscopy* ... (6) 5th Ed. 1888
**Jennings, C. E.** ... *Cancer* ... (6) 1889
**Jessett, F. B.** ... *Cancer of the Mouth, Tongue, and Alimentary Tract* (6) 1886
,, ... *Cancer of the Uterus* ... (6) 1894
**Jewett, C.** ... *Childbed Nursing* ... (6) 1891
*Kallos: A Treatise on the Scientific Culture of Personal Beauty and the Cure of Ugliness* ... (6) 1883
**Keetley, C. B.** ... *On the Surgery of the Knee-Joint* ... (6) [N.D.]

**Keith, T. and S.** ... *Contributions to the Surgical Treatment of Tumours of the Abdomen.* 2 parts... ... ... ... ...(6) 1885-89

**Kellgren, A.** ... *Technic of Manual Treatment* ... ... ... ... (6) 1890

**Kelynack, T. N.**... *Pathology of the Vermiform Appendix* ... ... ... (6) [1893]

**Kenwood, H. R.** ... *Essentials of Medical Anatomy* ... ... ... ... (6) 1889

**Kerr, J. G. D.** ... *Guide to the Bath Waters* ... ... ... ... ... (6) [1884]

**Keyes, W. H. Van Buren and E. L.** *Genito-Urinary Diseases and Syphilis* (6) 1881

**Kinsey-Morgan, A.** *Bournemouth as a Health Resort* ... ... ... ... (6) 1889

**Klebs, E.** ... ... *Die Allgemeine Pathologie.* Theil I. ... .. ... (6) 1887

**Kraus, J.** ... ... *Carlsbad* ... ... ... ... ... ... ... ... (6) 3rd Ed. 1887

**Kühne, H.** ... ... *Bacteria in Animal Tissues.* (Tr. and Ed. by V. D. Harris) ... ... ... ... ... ... ... ... (6) 1890

**Laffan, T.** ... ... *The Medical Profession, 1887* ... ... ... ... (6) 1888

**Lawson, R.** ... ... *The Milroy Lectures* ... ... ... ... ... ... (6) 1888

**Leuckart, R.** ... *The Parasites of Man.* (Tr. by W. E. Hoyle) ... (6) 1886

*Lexicon of Medicine and the Allied Sciences, The New Sydenham Society's.* Part XXIII. Puke—Scap. ... ... ... ... 1897

*Leysin : A Sanatorium and a High Altitude Health Resort* ... ... ... (6) 1893

**Lilley, G. H.** ... *The Tourist's Guide to the Isle of Portland* ... ... (6) 1883

**Lindsay, J. A.** ... *The Climatic Treatment of Consumption* .. ... (6) 1887

**Lindsay, W. L.** ... *Mind in the Lower Animals.* 2 vols ... ... ... (6) 1879

**Little, W. J. and E. M.** *On In-Knee Deviation* ... ... ... ... ... (6) 1882

**Lockwood, C. B.** ... *On Hernia* ... ... ... ... ... ... ... ... (6) 1889

**Loomis and W. G. Thompson, A. L.** [Eds.] *A System of Practical Medicine.* Vols. I., II. ... ... ... ... ... ... 1897

**Lubet-Barbon, A. Broca and F.** *Mastoid Abscesses and their Treatment.* (Tr. and Ed. by H. J. Curtis) ... ... ... 1897

**Lund, E.** ... ... *Hunterian Lectures,* 1885 ... ... ... ... ... (6) 1886

**Macewen, W.** ... *Osteotomy* ... ... ... ... ... ... ... ... ... (6) 1880

**Macfarlane, A. W.** *Insomnia and its Therapeutics* ... ... .. ... (6) 1890

**Mackay, J. Cleland and J. Y.** *Human Anatomy* ... ... ... ... ... ... 1896

**McKay, W. J. S.**... *Lawson Tait's Perineal Operations* ... ... ... ... 1897

**M'Kendrick, J. G.**.. *A Text-Book of Physiology.* 2 vols. ... ... ...(6) 1888-89

**Mackenzie, M.** ... *Hoarseness, Loss of Voice, and Stridulous Breathing* (6) 2nd Ed. 1868

„ ... *Diseases of the Throat and Nose.* Vol. II. ... ... (6) 1884

„ ... *Hay Fever* ... ... ... ... ... ... ... (6) 4th Ed. 1887

**McMurtry, L. S.**... *Nursing in Pelvic Surgery* ... ... ... ... ... (6) 1894

**Macnamara, C.** ... *Diseases of the Bones and Joints* ... ... (6) 2nd Ed. 1881

**Macpherson, J.** ... *Bath, Contrexéville, and the Lime Sulphated Waters* (6) 1886

**Maddox, E. E.** ... *Ophthalmological Prisms* ... ... ... ... .. (6) 1889

**Mahlendorff, P.** ... *The Science and Art of Adjustment between the Producing and Reflecting Vocal Apparatus* ... ... ... 1897

**Marshall, C. F.** ... *Elementary Physiology for Nurses* ... ... ... ... 1897

**Mason, F.** ... ... *The Study of the Face* ... ... ... ... ... ... (6) 1878

**Maunsell, S. E.** ... *Medical Experiences in India* ... ... ... ... (6) 1885

**Mayhew, [—]** ... *Illustrated Horse Management.* (Ed. by J. I. Lupton) (6) 1876

**Middleton, J.** ... *Lithotomy* ... ... ... ... ... ... ... ... (6) 1727

**Minor, T. C.** ... *Athothis: A Satire on Modern Medicine* ... ... (6) 1887

*Montreal, Official Guide to* ... ... ... ... ... ... ... ... ... ... (7) 1897

**Morgan, C. L.** ... *Animal Biology* ... ... ... ... ... ... ... (6) 1887

**Morris, H.** ... ... *Anatomy of the Joints* ... ... ... ... ... ... ... (6) 1879
,, ... *Surgical Diseases of the Kidney* ... ... ... ... (6) 1885
**Morten, Honnor**... *The Midwives' Pocket-Book* ... ... ... ... ... ... ... 1897
**Morton, J.** ... ... *Spina Bifida* ... ... ... ... ... ... (6) [2nd Ed.] 1887
**Moure, E. J.** ... *Sur trois Cas de Complications intra-craniennes d'Origine otique* ... ... ... ... ... ... ... ... ... 1897
**Murrell, W.** ... *Massage* ... ... ... ... ... ... ... ... (6) 3rd Ed. 1887
,, ... *Massotherapeutics* ... ... ... ... ... ... (6) 5th Ed. 1890
*Nederlandsch Tijdschrift voor Geneeskunde—Feestbundel Donders-Jubiléum* (6) 1888
**Newman, D.** ... *On Malpositions of the Kidney* ... ... ... ... ... (6) 1883
**Norris and C. A. Oliver, W. F.** [Eds.] *System of Diseases of the Eye.* Vols. I., II. ... ... ... ... ... ... ... 1897
**Ogle, J. W.** ... ... *The Relief of Tympanites by Puncture of the Abdomen* (6) 1888
**Oliver, W. F. Norris and C. A.** [Eds.] *System of Diseases of the Eye.* Vols. I., II. ... ... ... ... ... ... ... ... ... 1897
**Oliver, G.** ... ... *Bedside Urine-Testing* ... ... ... ... (6) 4th Ed. 1889
**Osler, W.** ... ... *Abdominal Tumors* ... ... ... ... ... ... ... (6) 1895
*Ottawa* ... ... ... ... ... ... ... ... ... ... ... ... ... ... (7) 1897
**Owen, E.** ... ... *Selected Subjects in connection with the Surgery of Infancy and Childhood* ... ... ... ... ... ... ... (6) 1890
**Paget, J.** ... ... *Lectures on Surgical Pathology.* 2 vols. ... ... (6) 1853
**Paget, S.** ... ... *John Hunter* (1728—1793) ... ... ... ... ... ... ... 1897
*Pamphlets.* 26 vols. ... ... ... ... ... ... ... ... ... ... ... ... ... (6) [V.Y.]
**Parish, E.** ... ... *Hallucinations and Illusions* ... ... ... ... ... ... ... 1897
**Park, R.** ... ... *An Epitome of the History of Medicine*... ... ... ... 1897
**Parker, R. W.** ... *Tracheotomy in Laryngeal Diphtheria* ... (6) 2nd Ed. 1885
,, ... *Congenital Club-Foot* ... ... ... ... ... ... ... (6) 1897
**Parkes, L. C.** ... *Hygiene and Public Health* ... ... ... ... 5th Ed. 1897
**Parry, J. S.** ... *Extra-Uterine Pregnancy* ... ... ... ... ... ... (6) 1876
**Péan, J.** ... ... *Diagnostic et Traitement des Tumeurs de l'Abdomen et du Bassin.* 3 vols. ... ... ... ... ... ... ... (6) 1895
**Percival, T.**... ... *Medical Ethics* ... ... ... ... ... ... ... ... (6) 1803
**Pettigrew, T. J.**... *Medical Portrait Gallery.* 4 vols. in 2 ... ... (6) 1840
*Pharmacopœia of the Bristol Royal Infirmary* ... ... ... ... ... ... ... (6) 1871
*Pharmacopœia of the Central London Throat, Nose, and Ear Hospital* ... (6) 1890
*Pharmacopœia, The Pocket A B C Materia Medica and Modern* ... ... (6) 1888
**Phillips, J.** ... ... *Outlines of the Diseases of Women* ... ... 2nd Ed. 1897
**Pick, T. P.** ... ... *Fractures and Dislocations* ... ... ... ... ... ... (6) 1885
**Poore, G. V.**... ... *Nervous Affections of the Hand, and other Studies* ... 1897
**Poulet, A.** ... ... *Foreign Bodies in Surgery.* 2 vols. ... ... ... (6) 1881
**Powell, Rev. B.** ... *Unity of Worlds* ... ... ... ... ... ... ... ... (6) 1855
**Power, W. J. Walsham and D'A.** *Surgical Pathology* ... (6) 2nd Ed. 1890
**Pozzi, S.** ... ... *Traité de Gynécologie* ... ... ... ... ... ... ... (6) 1890
**Predöhl, A.** ... *Die Geschichte der Tuberkulose* ... ... ... ... ... (6) 1888
**Priestley, W. O.**... *The Pathology of Intra-Uterine Death* ... ... ... (6) 1887
**Prochownick, L.**... *Massage in der Frauenheilkunde* ... ... ... ... (6) 1890
**Pye, W.** ... ... *Children's Deformities* ... ... ... ... ... ... ... (6) [1889]
,, ... ... *Elementary Bandaging and Surgical Dressing* (6) 3rd Ed. 1889
**Quain, R.** [Ed.]... *A Dictionary of Medicine.* 2 parts ... ... ... (6) 1883

| | | | |
|---|---|---|---|
| **Rabagliati, A.** | *Classification and Nomenclature of Diseases* | (6) | 1886 |
| ,, | *Air, Food, and Exercises* | | [N.D.] |
| **Rainy, R. Hutchison and H.** | *Clinical Methods* | | 1897 |
| **Ralfe, C. H.** | *Diseases of the Kidneys* | (6) | 1885 |
| **Randolph, G.** | *On the Bristol Water* | (6) | 1745 |
| **Reeves, H. A.** | *Practical Orthopædics* | (6) | 1885 |
| **Ribot, T.** | *The Psychology of the Emotions* | | 1897 |
| **Ringer and H. Sainsbury, S.** | *Hand Book of Therapeutics* | 13th Ed. | 1897 |
| **Riverius, L.** | *The Practice of Physick* | (6) | 1678 |
| **Rivington, W.** | *Rupture of the Urinary Bladder* | (6) | 1884 |
| ,, | *The Medical Profession, 1887* | (6) | 1888 |
| **Roberts, J. B.** | *Surgical Delusions and Follies* | (6) | 1884 |
| ,, | *Operative Surgery of the Human Brain* | (6) | 1885 |
| **Roberts, R. L.** | *Ambulance Work* | (6) 3rd Ed. | 1888 |
| **Robinson, T.** | *Syphilis* | (6) | 1886 |
| **Robinson, W.** | *Endemic Goitre* | (6) | 1885 |
| **Robson, A. W. M.** | *Instruments and Appliances required in Operations* | (6) | 1889 |
| **Rogers, W. T.** | *A Manual of Bibliography* | New Ed. | 1891 |
| **Roose, R.** | *Leprosy* | (6) | 1890 |
| **Ross, J.** | *Diseases of the Nervous System* | (6) | 1885 |
| *Royal Medical and Chirurgical Society of London, Charter and Bye-Laws of the* | | (6) | 1882 |
| **Rühl, W.** | *Die Anatomie und Behandlung der Geburtsstörungen* | | 1897 |
| **Rutty, W.** | *A Treatise of the Urinary Passages* | (6) | 1726 |
| **S., R. S.** | *The Complete Guide to Clevedon and its Vicinity* | (6) | [N.D.] |
| **Sainsbury, S. Ringer and H.** | *Hand Book of Therapeutics* | 13th Ed. | 1897 |
| *Salernum—De Conservanda Bona Valetudine* | | (6) | 1562 |
| **Salisbury, J. H.** | *The Salisbury Treatment of Disease* | (6) | 1887 |
| **Sanderson, J. B.** | *Handbook of the Sphygmograph* | (6) | 1867 |
| **Savage, G. H.** | *Insanity* | (6) | 1884 |
| **Schech, P.** | *Diseases of the Mouth, Throat, and Nose.* (Tr. by R. H. Blaikie) | (6) | 1886 |
| **Schmiegelow, E.** | *Asthma* | (6) | 1890 |
| **Schultze, B. S.** | *Displacements of the Uterus.* (Tr. by J. J. Macan and Ed. by A. V. Macan) | (6) | 1888 |
| **Senn, N.** | *The Surgical Treatment of Intestinal Obstruction* | (6) | 1888 |
| **Service, J.** | *The Feeding and Care of Infants* | (6) | 1890 |
| **Severn, E. F. Grün and W. D.** | *Handbook to Dr. Koch's Treatment in Tubercular Disease* | (6) | [1890] |
| **Sharp, W.** | *Therapeutics founded upon Organopathy and Antipraxy* | (6) | 1886 |
| **Shaw, J.** | *Antiseptics in Obstetric Nursing* | (6) | 1890 |
| **Shiercliff, E.** | *Bristol and Hotwell Guide* | (6) 1789; (6) 4th Ed. | [N.D.] |
| **Simon, J.** | *Lectures on Pathology* | (6) | 1850 |
| **Simon, R. M.** | *Treatment of Skin Diseases* | (6) | 1888 |
| **Sinclair, W. J.** | *Gonorrhœal Infection in Women* | (6) | 1888 |
| **Smith, E. N.** | *The Surgery of Deformities* | (6) | 1882 |
| ,, | *Curvatures of the Spine* | (6) 3rd Ed. | 1889 |
| ,, | *Spinal Caries* | 2nd Ed. | 1897 |
| **Smith, F. W.** | *The Leamington Waters* | (6) | 1884 |

**Smith, J. G.** ... *Abdominal Surgery* (6) 1887; (6) 2nd Ed., 1888; (6) 3rd Ed., [1889]; (6) 4th Ed. [1891]
„ ... *Chirurgie abdominale.* (Tr. par P. Vallin) ... (6) 1894
**Smith, R. M.** ... *The Physiology of the Domestic Animals* ... ... (6) 1889
**Smits, J. C. J. C.**... *Vergleichende Beurtheilung der Verschiedenen Methoden des Steinschnitts* ... ... ... ... ... ... (6) 1887
**Snow, H.** ... ... *Clinical Notes on Cancer*... ... ... ... ... ... (6) 1883
„ ... ... *The General Theory of Cancer-Formation* ... ... (6) 1889
„ ... ... *Re-appearance of Cancer* ... ... ... ... ... (6) 1890
„ ... ... *The Palliative Treatment of Incurable Cancer* ... (6) 1890
**Spiegelberg, O.** ... *A Text-Book of Midwifery.* (Tr. from 2nd German Ed. by J. B. Hurry) Vol. I. ... ... ... (6) 1887
**Spitzka, E. C.** ... *Insanity* ... ... ... ... ... ... ... ... ... (6) 1883
**Squire, P.** ... ... *Pharmacopœias of Twenty-two of the London Hospitals* (6) 3rd Ed. 1874
**Stanley, E.** ... ... *Diseases of the Bones* ... ... ... ... ... ... (6) 1849
**Stanton, Mary O.** *A System of Practical and Scientific Physiognomy.* 2 vols. ... ... ... ... ... ... ... ... (6) 1890
**Starr, L.** ... ... *Diseases of the Digestive Organs in Children* ... (6) 1886
**Steven, J. L.** ... *Practical Pathology* ... ... ... ... ... ... (6) 1887
**Stevenson, W. F.**.. *Wounds in War* ... ... ... ... ... ... ... ... 1897
**Stewart, W. R. H.** *Epitome of Ear Diseases* ... ... ... ... ... (6) 1888
**Stoker, G.** ... ... *The Oxygen Treatment for Wounds, Ulcers, Burns,* [*&c.*] 1897
**Strümpell, A.** ... *A Text-Book of Medicine.* (Tr. from 2nd and 3rd German Ed. by H. F. Vickery and P. C. Knapp) (6) 1887
*Surgery, An American Text-Book of.* (Ed. by W. W. Keen and J. W. White) ... ... ... ... ... ... ... ... (6) 1892
**Sutton, J. B.** ... *Ligaments* ... ... ... ... ... ... ... ... (6) 1887
„ ... *Dermoids* ... ... ... ... ... ... ... ... ... (6) 1889
**Swieten, G. van** ... *Commentaria in H. Boerhaave Aphorismos* (6) Tomes I., II., 3rd Ed., 1752–59, III., 2nd Ed., 1755, IV., V. 1764–72
**Tait, L.** ... ... *Diseases of the Ovaries* ... ... ... ... (6) 4th Ed. 1883
„ ... ... *Lectures on Ectopic Pregnancy and Pelvic Hematocele* (6) 1888
**Taylor, A.** ... ... *The Sanitary Inspector's Handbook* ... ... 2nd Ed. 1897
**Taylor, H. C.** ... *Wanderings in Search of Health* ... ... ... ... (6) 1890
**Taylor, S.** ... ... *Sound and Music* ... ... ... ... ... ... ... (6) 1873
**Teale, T. C. Allbutt and T. P.** *Clinical Lectures* ... ... ... ... ... (6) 1885
**Thin, G.** ... ... *Cancerous Affections of the Skin* ... ... ... ... (6) 1886
[**Thomas, H. O.**] ... *The Collegian of 1666 and the Collegians of 1885*.. (6) 1885
**Thompson, E. S.**... *Influenza: An Historical Survey* ... ... ... ... (6) 1890
**Thompson, Sir H.**.. *Stricture of the Urethra* ... ... ... ... (6) 4th Ed. 1885
„ ... *Diseases of the Prostate* ... ... ... ... (6) 6th Ed. 1886
„ ... *The Suprapubic Operation of Opening the Bladder* (6) 1886
**Thompson, A. L. Loomis and W. G.** [Eds.] *A System of Practical Medicine.* Vols. I., II. 1897
**Thornton, J. K.** ... *Surgery of the Kidneys* ... ... ... ... ... ... (6) 1890
**Tibbits, H.** ... ... *How to Use a Galvanic Battery* ... ... (6) 3rd Ed. 1886
„ ... *Massage* ... ... ... ... ... ... ... (6) 2nd Ed. 1888
**Tosswill, L. H.** ... *Diseases of the Eye* ... ... ... ... ... ... ... (6) 1884
*Tourist Travel, Gateways of* ... ... ... ... ... ... ... ... ... ... (7) 1897
**Towgood, J. D.** ... *De Vipera* ... ... ... ... ... ... ... ... ... (6) 1718

**Toynbee, J.** ... ... *On the Organisation and Nutrition of Non-Vascular Animal Tissues* ... ... ... ... ... ... ... (6) 1814
**Treves, F.** ... ... *Scrofula and its Gland Diseases* ... ... ... ... ... (6) 1882
,, ... ... *Peritonitis* ... ... ... ... ... ... ... ... ... (6) 1894
**Tuke, D. H.** ... ... *The Insane in the United States and Canada* ... (6) 1885
,, ... ... *Insane Poor in Yorkshire* ... ... ... ... ... ... (6) 1889
**Ultzmann, R.** ... *On Sterility and Impotence in Man.* (Tr. by A. Cooper) (6) 1887
**Vacher, F.** ... ... *Defects in Plumbing and Drainage Work* ... ... (6) 1889
**Van Buren and E. L. Keyes, W. H.** *Genito-Urinary Diseases with Syphilis* (6) 1881
**Wallace, A. R.** ... *Travels on the Amazon and Rio Negro.* (Ed. by G. T. Bettany) ... ... ... ... ... ... ... ... (6) 1889
**Walsham, W. J.** ... *Surgery* ... ... ... ...(6) 5th Ed., 1895; 6th Ed. 1897
,, **and D'A. Power.** *Surgical Pathology* ... ... (6) 2nd Ed. 1890
**Weatherly, L. A.**. *Ambulance Lectures* ... ... ... ... ... ... ... (6) [1880]
,, ... *Lectures on Domestic Hygiene and Home Nursing* (6) [1880]
,, ... *The Care and Treatment of the Insane in Private Dwellings* ... ... ... ... ... ... ... ... (6) 1882
**Weaver, J.** ... ... *Pulmonary Consumption*... ... ... ... ... ... ... (6) 1887
**Webster, J. C.** ... *Ectopic Pregnancy* ... ... ... ... ... ... ... ... (6) 1895
**Weisse, F. D.** ... *Practical Human Anatomy* ... ... ... ... ... (6) 1886
**Wells, Sir S.** ... *Diagnosis and Surgical Treatment of Abdominal Tumours* ... ... ... ... ... ... ... ... ... (6) 1885
**West, C.** ... ... *The Mother's Manual of Children's Diseases* ... (6) 1885
**Westcott, W. W.**... *Suicide* ... ... ... ... ... ... ... ... ... ... ... (6) 1885
**Wharton and B. F. Curtis, H. R.** *The Practice of Surgery*... ... ... ... 1898
**White, W. H.** ... *The Means by which the Temperature of the Body is maintained in Health and Disease* ... ... ... 1897
**Whitney, W. D.** ... *The Life and Growth of Language* ... ... ... (6) 1875
**Whittle, E. G.** ... *Congestive Neurasthenia* ... ... ... ... ... ... (6) 1889
**Williams, J.** ... *Cancer of the Uterus* ... ... ... ... ... ... ... (6) 1888
**Williams, W. R.**... *The Influence of Sex in Disease* ... ... ... ... (6) 1885
,, ... *The Principles of Cancer and Tumour Formation*... (6) 1888
**Wise, A. T.** ... ... *Alpine Winter in its Medical Aspects* ... (6) 2nd Ed. 1885
**Witkowski, G.-J.**.. *La Génération humaine* ... ... ... ... ... 7th Ed. [N.D.]
,, ... *Histoire des Accouchements chez tous les Peuples* [N.D.]
,, ... Do. Appendice—*L' Arsenal obstétrical* [N.D.]
,, ... *The Evil that has been said of Doctors.* (Tr. by T. C. Minor) ... ... ... ... ... ... ... ... ... (6) 1889
**Woakes, E.** ... ... *Nasal Polypus*... ... ... ... ... ... ... ... (6) 1887
**Woodberry, G. E.**. *A History of Wood-Engraving* ... ... ... ... (6) 1883
**Woodhead, G. S.**... *Practical Pathology* ... ... ... ... ... (6) 2nd Ed. 1885
**Worthington, L.-N.** *Thérapeutique. Ligatures des Artères. Trachéotomie et Laryngotomie* ... ... ... ... ... ... (6) 1889
**Yearsley, J.** ... *Throat Ailments* ... ... ... ... ... (6) 8th Ed. 1867
**Young, D.** ... ... *Rome in Winter and the Tuscan Hills in Summer* (6) 1886

### TRANSACTIONS, REPORTS, JOURNALS, &c.

Academy of Medicine in Ireland, Transactions of the (6) Vols. II., III., 1884–85, VI. 1888
American Association of Obstetricians and Gynecologists, Transactions of the (6) Vols. I.–VII., 1888–95, IX. 1897

American Gynæcological and Obstetrical Journal, The ... ... Vol. X. 1897
American Journal of Obstetrics, The. ... Vols. (2) XVI., 1883; (5) XXI. 1888
American Medical Digest, The ... ... ... ... ... ... ... (5) Vol. IV. 1885
American Pediatric Society, Transactions of the ... ... Vol. VIII. 1896
American Practitioner, The ... ... ... (6) [Vols. XXX.-XXXII.] 1884-85
Annual of the Universal Medical Sciences. 5 vols. ... ... ... ... 1896
Association of American Physicians, Transactions of the Vol. XII. 1897
Australasian Medical Gazette, The ... ... ... ... ... Vol. VII. 1888
Australian Medical Journal, The ... ... ... ... ...(6) N.S., Vol. XI. 1889
Birmingham Medical Review, The ... ... (6) Vols. XXIII., XXIV. 1888
Buffalo Medical Journal ... ... ... ... ... ... ... Vol. XXXVI. 1897
Cincinnati Lancet-Clinic, The ... ... ... ... ... ... Vol. LXVI. 1891
Clinical Journal, The ... ... ... ... ... ... ... ... ... Vol. X. 1897
Clinical Society's Transactions ... ... ... ... ... ... Vol. XXX. 1897
Congrès international de Médecins des Colonies, Amsterdam, Sept. 1883,
Compte-Rendu ... ... ... ... ... ... ... ... ... ... ... ... (6) 1884
Directory, Bristol and Clifton ... ... ... ... ... ... ... (6) 1874, 1891
Directory of Bristol, Kelly's ... ... ... ... ... ... ... ... ... (6) 1883
Edinburgh Clinical and Pathological Journal, The ... ... (6) Vol. I 1884
Edinburgh Medical Journal, The. (6) Vols. XXXII., Part 2,
XXXIII., Part 1 1887-88
Gazette de Gynécologie ... ... ... ... ... ... (6) Tomes I.-IV. 1886-89
General Medical Council, Minutes of the ... ... Vols. I.-VIII. 1863-71
Hazell's Annual ... ... ... ... ... ... ... ... ... ... ... ... ... (6) 1892
Hospital, The ... ... ... ... ... ... ... ... ... ... ... Vol. XXII. 1897
Hospitals—
Medical and Surgical Report of the Children's Hospital, [Boston],
1869-1894 ... ... ... ... ... ... ... ... ... ... (6) 1895
Royal Victoria Hospital, Montreal, The. 3rd Annual Report for
1896... ... ... ... ... ... ... ... ... ... ... ... (7) [1897]
Illustrirte Monatsschrift der ärztlichen Polytechnik 1886; (6) 1888; (6) 1889
Indian Medical Journal, The ... ... ... ... ... ... ... (6) Vol. V. 1886
Intercolonial Medical Congress of Australasia, Transactions of Second
Session ... ... ... ... ... ... ... ... ... ... ... ... ... (6) 1889
International Clinics ... ... ... ... ... ... (6) 3rd Ser., Vol. I. 1893
Journal of Cutaneous and Venereal Diseases ... ... ... (4) Vol. III. 1885
Journal of Mental Science, The. (3) Vols. XXIII., XXIV., 1878-79,
XXXVIII., 1892, XL. 1894
Laboratory Reports. Royal College of Physicians, Edinburgh.
Vols. V., VI. 1894-97
McGill College and University, Annual Calendar of, 1897-98 ... ... (7) 1897
McGill University, Calendar of the Faculty of Medicine, 1896-97 (7) 1896
Medical Act (1858) Amendment (No. 3) Bill. Report ... ... ... (6) 1880
Medical and Surgical Reporter ... ... ... ... ... ...Vol. LXXVI. 1897
Medical Chronicle, The ... ... ... ... ... ... ... N.S., Vol. VII. 1897
Medical News, The ... ... ... ... ... ... ... (5) Vols. XL., XLI. 1882
Medical Record, The ... ... ... ... ... (5) Vols. XVI.-XVIII. 1879-80
Medico-Chirurgical Transactions ... ... ... ... ... ... Vol. LXXX. 1897
Memoria de los Trabajos ejecutados por el Consejo Superior de Salubridad en el Año de 1895 ... ... ... ... ... ... ... ... ... ... ... 1897
Memphis Medical Monthly, The. (5) Vols. IX., X., 1889-90; XIII., XIV. 1893-94
Michigan State Medical Society, Transactions of the .. Vol. XXI. 1897
Montreal Medical Journal, The ... ... ... ... ... (6) Vol. XVII. 1889
New Hampshire Medical Society, Transactions of the ... ... ... ... 1897
Nouvelles Archives d'Obstétrique et de Gynécologie... ... ... (6) 1886-88
Nursing Directory, Burdett's Official ... ... ... ... ... ... ... ... 1898
Obstetric Gazette, The ... ... Vols. (6) VIII.-XII., 1885-89; XIII. 1890
Pacific Medical and Surgical Journal ... (6) Vols. XXVIII.-XXXI. 1885-88
Perthshire Medical Association, The Transactions of the (6) Vol. I. 1882
Philadelphia County Medical Society, Proceedings of the Vol. XVII. 1896
Philadelphia Medical Times ... ... ... ... (6) Vols. XV.-XVII. 1885-87
Practitioner, The ... (6) Vols. XXXVI.-XXXIX., 1886-87, XLIII. 1889

Quarterly Medical Journal, The ... ... ... ... ... ... Vol. V. 1896-97
Répertoire universel d'Obstétrique et de Gynécologie ... ... (6) 1886-88
Satellite, The ... ... ... ... ... ... ... ... ... Vols. I.–III. 1887-90
University Medical Magazine... ... ... ... ... ... ... ... ... Vol. IX. 1897
Whitaker's Almanack ... ... ... ... ... ... ... ... ... ... (4) 1883
Year-Book of Scientific and Learned Societies ... ... ... ... ... (6) 1886

### Local Medical Notes.

### BATH.

The Roman Bath.—Of the triple function that H.R.H. the Duke of Cambridge took part in on October 18th, the ceremony of chief interest to us was the opening as a promenade the old Roman bath and a new concert room in connection with the pump room.

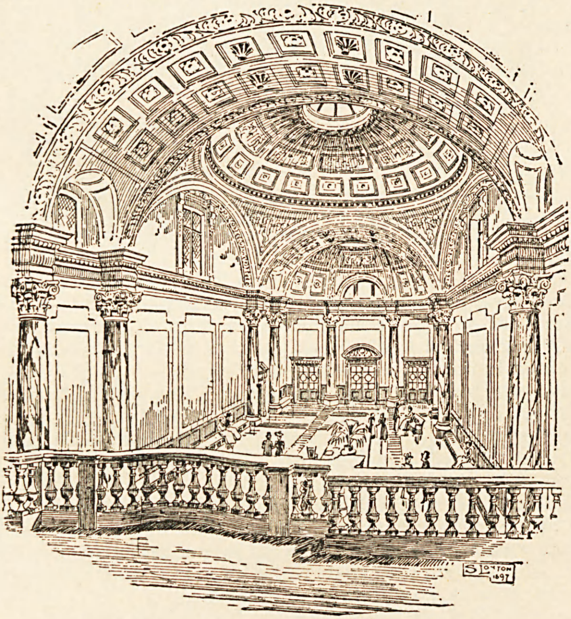

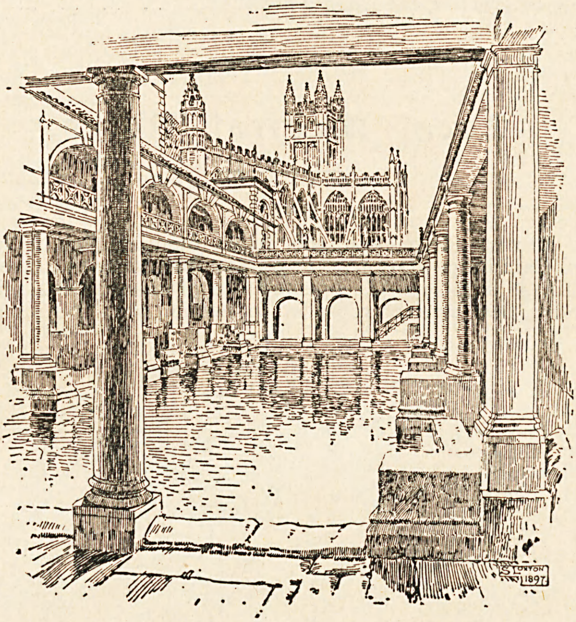

The Roman bath is of great archæological interest, and when complete probably extended over six acres of ground, so that the present restoration is only a small part of the magnificent building that existed in the first centuries of the Christian era. It is probable that soon after the recall of the Imperial troops the Britons destroyed as much as they could of the buildings that had been erected by the army of occupation, and it was not till 1754 that the ruins were discovered at a depth of fifteen feet below the present road level. In 1871 a much more extensive discovery was made, and a few years later the enterprising action of the Corporation in purchasing the property enabled still further excavations to be made. The present restoration has been, to our mind, very skilfully done: the characteristic features of the Roman style have been well preserved, without the profuse ornamentation that was so conspicuous in later Roman work. Having been built originally about A.D. 54, it is considerably older than many of the ruined baths in the Eternal City itself.

It was peculiarly apt that the opening day should have been fixed for St. Luke's day; and though the huge crowd that was present somewhat interfered with a critical inspection, it was evident that the city of Bath had added another and a very substantial attraction for visitors, whether patients or not.

On the same evening a dinner was given by the medical profession of Bath to the visiting doctors. To say that it was a success would be to damn it with faint praise. The chair was taken by Dr. Weatherly, who in a brief speech said that, while he hoped and believed that the baths were now as complete and perfect as any to be found on the Continent or elsewhere, he felt assured that if the patients who appear to believe that no baths or hydropathic treatment at home could equal those abroad would only visit Bath, they would find not only the appliances better, the water as efficacious and the medical skill as great, but also the surroundings as delightful as those to be found in any spa in the world.

Obituary.—The medical profession in Bath have to regret the death of Mr. H. W. Freeman, which took place at his residence, 24 The Circus, on November 28th, from cardiac failure. His health had been weak for some time, but it was not until shortly after the opening of the New Pump Room in October that he became seriously unwell.

He was born near Westward Ho! in 1842, and after being educated at the Bideford Grammar School he went to the Middlesex Hospital, where he studied with much success, obtaining prizes in surgery and medicine. In 1864 he qualified as M.R.C.S. Eng., as L.R.C.P. Lond. in 1865, and as F.R.C.S. Irel. in 1882. In 1864 he was appointed Resident Medical Officer to the Royal United Hospital, and about a year afterwards commenced to practise in Gay Street, whence he soon migrated to The Circus. He soon became known to a wide circle, and acquired a large surgical practice.

In 1881 Mr. Freeman was appointed Honorary Surgeon to the Hospital, and at his death still held this post. In 1887 he was elected to the Town Council, and in the following year was appointed Mayor. During his term of office the new King's and Queen's baths were opened by H.R.H. the Duchess of Albany. At the expiration of his Mayoralty he presented to the Corporation a beautiful statue, "The Angel at the Pool," which now stands over the fountain in the pump-room. In 1893 he was placed on the Commission of the Peace for the City, and also for the County of Somerset. For many years he devoted much time to the development of the baths, and quite recently, at his suggestion, the Nauheim system was introduced. In 1887 he most generously offered a house on Lansdown to the city for a Convalescent Home, but unfortunately, through lack of endowment funds, the scheme fell through.

Mr. Freeman's principal literary efforts were *The Thermal Baths of Bath*, published in 1888, and *New Methods of Cure at Bath*, published only last month. His one hobby was horses, and his extensive stud at Weston made him well known to all lovers of the horse, not only in this country but abroad. He leaves a widow, for whom great sympathy is felt. His death will be a great loss, not only to the profession, but to many who had known and respected him, and to the city for which —public-spirited citizen as he was—he had done so much.

### BRISTOL.

Civic Honours.—Bristol medical men appear to be sharing in the civic changes which occur about this time of the year, and no doubt the profession will congratulate itself as well as the new High Sheriff, Mr. Richardson Cross, and Mr. Hancocke Wathen, who has been made an Alderman. Mr. Colston Wintle was elected a member of the Town Council.

University College.—The following students have recently passed examinations:

**University of London.**—*Final Examination for M.B. Degree*—A. S. Cobbledick.

**Examining Board in England by the Royal Colleges of Physicians and Surgeons.**—*First Examination. Part III. Elementary Biology*—R. V. James. *Second Examination. Anatomy and Physiology*—L. A. Moore. *Final Examination. Surgery*—C. M. Mitchell, R. J. Rogers*; *Medicine*—R. J. Rogers,* J. E. Long; *Midwifery*—W. J. Lord, E. V. Foss.

* Completes Examination.

The Typhoid Outbreak.—A supply of tubes for use in the blood-serum test is kept by Messrs. Ferris & Co., Union Street, and Messrs. Buxton & Co., Queen's Road. Tubes can also be obtained direct from the Medical Officer of Health, who is prepared, upon receipt of a small quantity of the patient's blood, to apply the test and furnish the doctor with a report.

At the Meeting of the Bristol Medico-Chirurgical Society on December 8th, Dr. E. Long Fox spoke of the assistance that Dr. D. S. Davies, the Medical Officer of Health, and Mr. J. C. Heaven, the Assistant Medical Officer, had given to the profession during the present epidemic of enteric fever. Dr. Fox referred in well-chosen words to the high estimation in which both these gentlemen were held, and said that the profession of Bristol could not let the opportunity pass without expressing their gratitude to them for their tact, courtesy, and assistance. Dr. Shaw, the president, then presented to each of these gentlemen an Album containing the following, with the names of 172 local doctors appended to it:

> "Dear Sir.—The Medical Profession of Bristol and Clifton are anxious to express to you their sense of the services you have rendered in the late epidemic of Typhoid Fever. The acumen shewn in tracing the source of the epidemic, the precautions taken for limiting the mischief, the constant assistance given to the profession at large, and the masterly report on the whole matter, have laid the public and the medical profession under deep obligation to you, and prove beyond all question that Bristol is fortunate in commanding the services of Officers of Health who are keenly alive to the necessity for constant vigilance, and who will not spare themselves in carrying out with the greatest energy and tact whatever measures may be necessary for the public benefit."

Dr. Davies and Mr. Heaven suitably responded, and expressed their gratification at the presentation, but said they had done only that which it was their duty to do. Mr. Alderman Wathen said that the address was not presented as an acknowledgment of duty done, but as a recognition of the urbanity and special intelligence which the Medical Officers had displayed during the whole course of the outbreak.

Convalescent Home.—The cash in hand and promised for the Jubilee Convalescent Home amounts to £82,000. The idea at present is to spend about £20,000 of this on building, furnishing, &c., leaving the balance for endowment of 65 beds. These beds will be distributed as follows: to the Royal Infirmary, 30; the General Hospital, 30; the Eye Hospital, 1; the Bristol Dispensary, 3; and the Clifton Dispensary, 1. The sum also will, it is hoped, help to lessen the cost of the 15 other beds set apart for paying patients. The committee have worked well, and it is believed that the Jubilee Convalescent Home will for ever exist as a fitting memorial of the prosperous and happy period that this country enjoyed under the reign of Her Most Gracious Majesty, Queen Victoria.

---

**Alvarenga Prize of the College of Physicians of Philadelphia.**—The next award of the Alvarenga Prize will be made on July 14th, 1898. Essays intended for competition may be upon any subject in Medicine, and must be received by the Secretary of the College on or before May 1st, 1898. Further information may be obtained from the Secretary.

### LIST OF SUBSCRIBERS

TO

### The Bristol Medico-Chirurgical Journal.

*DECEMBER*, 1897.

---

### England.

### CHESHIRE.

NEW BRIGHTON.
A. W. Riddell

### CORNWALL.

EAST LOOE.
L. F. Houghton

FALMOUTH.
S. A. Lidiard

HELSTON.
B. C. Kendall

NEW QUAY.

A. Hardwick

PENZANCE.
W. S. Bennett
J. A. Fox
J. Symons

ROCHE.
W. R. Goodfellow

ST. AUSTELL.
W. Mason

### DEVONSHIRE.

BABBACOMBE.
T. Finch

BARNSTAPLE.
C. H. Gamble
J. Harper
M. Jackson

BRIXHAM.
G. C. Searle

BROAD CLYST.
J. Somer

BUDLEIGH SALTERTON.
T. G. C. Evans
H. F. Semple

CHAGFORD.
A. D. Hunt

CHUDLEIGH.
C. L. Cunningham

CULLOMPTON.
G. G. Gidley

DARTMOUTH.
R. B. Searle

DEVONPORT.
F. E. Row

EXETER.
J. M. Ackland
A. G. Blomfield
H. Davy
E. J. Domville
C. Fenwick
R. V. Solly
E. B. Steele-Perkins
R. Thomas
L. H. Tosswill

HONITON.
T. W. Shortridge

ILFRACOMBE.
F. W. Langridge

MILLBROOK.
A. B. Cheves

MORTHOE.

E. J. Hollings

NEWTON ABBOT.
J. Culross

OTTERY ST. MARY.
W. A. H. Barrett

PAIGNTON.
J. Alexander

PLYMOUTH.
G. F. Aldous
R. H. Clay
Medical Society
E. B. Thomson
W. L. Woollcombe

PLYMPTON.
C. Aldridge
W. D Stamp

### DEVONSHIRE *(Continued).*

SIDMOUTH.

J. H. S. Pullin

SOUTH MOLTON.

H. J. Smyth

TOPSHAM.

J. N. Frood

TORQUAY.

H. Humphreys
W. Odell
R Pollard
W. Powell
Medical Society
C. H. Wade

WINKLEIGH.

J. H. Norman

### DORSETSHIRE.

BEAMINSTER

T. P. Daniel

DORCHESTER.

P. W. MacDonald

STURMINSTER NEWTON.

J. C. Leach

WEYMOUTH.

J. M. Lawrie

WIMBORNE.

A. J. H. Crespi

### ESSEX.

NEWPORT.

W. A. Smith

### GLOUCESTERSHIRE.

BRISTOL.

W. R. Ackland
G. Adams
J. Ambrose
C. Atchley
W. M. Barclay
T. G. L. Baretti
B. J. Baron
A. E. Blacker
A. F. Blagg
E. C. Board
C. W. J. Brasher
J. Broom
C. A. La T. Brough
F. St. J. Bullen
E. F. H. Burroughs
J. P. Bush
A. Carr
T. M. Carter
W. F. Carter
T. Carwardine
R. H. Chilton
J. M. Clarke
T. V. Coker
H. Cook
W. H. Cory
W. Cotton
W. Coulter
F. R. Cross
J. Dacre
D. S. Davies
H. F. Devis
N. C. Dobson
E. L. W. Dunbar
E. Eberle
F. H. Edgeworth
W. Elder
C. Elliott
J. Ewens
E. Fawcett
R. G. Fendick
J. L. Firth
T. Fisher
E. L. Fox
J. Freeman
W. J. Fyffe
E. B. Garland
T. T. Genge
W. C. Gent
D. S. Gerrish
A. N. G. Gibbs
W. G. Grace
J. S. Griffiths
L. M. Griffiths
W. B. T. Gubbin
C. D. G. Hailes
A. H. Haines
H. E. Harris
A. J. Harrison
W. H. Harsant
A. G. Hayman
C. A. Hayman
J. C. Heaven
C. K. C. Herapath
H. E. Hetling
H. Hill
T. Hill
General Hospital
G. A. Imlay
H. W. Kendall
F. P. Lansdown
R. G. P. Lansdown
A. E. A. Lawrence
E. L. Lees
W. Ligertwood
J. J. S. Lucas
P. W. McCrea
J. C. McWatters
H. Marshall
C. E. Matthews
T. O. Mayor
H. F. Mole
A. T. Morgan
C. A. Morton
G. T. Myles
B. G. Neale
W. N. Nevill
C. Newman
W. H. C. Newnham
T. D. Nicholson
C. O'Brien
A. Ogilvy
G. S. Page
G. Parker
J. H. Parry
J. St. J. G. Parsons
A. W. Peake
W. A. Perry
C. F. Pickering
H. I. Pocock
W. E. Pountney
J. J. Powell
A. Prichard
A. W. Prichard
J. E. Prichard
A. Pring
A. B. Prowse
M. Randall
B. M. H. Rogers
W. B. Roué
C. K. Rudge
H. T. Rudge
J. E. Shaw
E. J. Sheppard
E. M. Skerritt
G M. Smith
J. G. Smith
R. S. Smith
C. Steele
J. B. Stephenson
W. H. Stevens
F. W. Stoddart
J. Swain
J. G. Swayne
S. H. Swayne
W. C. Swayne
J. O. Symes
J. Taylor
W. J. Tivy
H. Waldo

### GLOUCESTERSHIRE *(Continued).*

BRISTOL *(continued).*

C. H. Walker
C. H. Wallace
J. H. Wathen
C. J. Whitby
J. Wilding
C. Williams
P. W. Williams
H. W. Windsor-Aubrey
C. Wintle

CHELTENHAM.

C. E. F. Mouat-Biggs
G. A. Peake
E. Trevithick
L. Winterbotham

CHIPPING SODBURY.

A. Grace

CIRENCESTER.

W. R. Cossham
O. H. Fowler
C. Mackinnon

COLEFORD.

L. B. Trotter

DOWNEND.

H. Skelton

FISHPONDS.

W. Brown

GLOUCESTER

R. W. Batten
A. H. Benson
W. R. Hadwen
J. G. Soutar

HAMBROOK.

E. Crossman
F. W. Crossman
W. F. B. Eadon

KINGSWOOD.

R. W. Brimacombe

LYDNEY.

W. P. Kennedy

MINCHINHAMPTON.

B. Church

PARKEND.

W. C. Halpin

REDFIELD.

J. W. Rodgers

STAPLE HILL.

W. Murray

STAPLETON.

H. A. Benham

STOKE BISHOP.

H. A. Beaver
C. E. Wedmore

STONEHOUSE.

G. T. B. Watters

STOW-ON-THE-WOLD

E. Dening

STROUD.

A. S. Cooke
E. A. Howsin

TETBURY.

W. Wickham

THORNBURY.

E. M. Grace
L. H. Williams

WESTBURY-ON-TRYM.

A. Harvey
H. L. Ormerod

WINTERBOURNE.

R. Eager

WOTTON-UNDER-EDGE

T. C. Boyce
D. H. Forty

### HAMPSHIRE.

ALDERSHOT.

J. C. Barr

ALTON.

W. L. P. Bevan

BOURNEMOUTH.

G. E. Crallan
J. S. Dickie
J. G. Harsant
J. Horsfall

CARISBROOKE.

J. Groves

DROXFORD.

A. H. Goodwyn

FLEET.

H. Wilcox

LYMINGTON.

J. Rendall

SOUTHAMPTON

W. R. Y. Ives

SOUTHSEA.

J. W. Cousins

VENTNOR.

E. R. Woodford

### HUNTINGDONSHIRE.

ST. NEOT'S.

T. C. Grey
J. H. Hemming

### KENT.

TUNBRIDGE WELLS.

W. J. K. Millard

### LANCASHIRE.

LIVERPOOL.

G. A. Hawkins-Ambler
Medical Institution

POULTON-LE-FYLDE.

P. H. Day

PRESTON

J. E. Garner

### MIDDLESEX.

LONDON.

J. Berry
J. F. Evans
T. C. Fox
M. Handfield-Jones
W. H. A. Jacobson
H. M. Jones
Lord Lister
E. J. Maclean
J. Macready
A. L. Marshall
F. T. Roberts
W. J. Seward
E. C. Taylor
F. J. Wethered
W. M. Willis
G. S. Woodhead

TOTTENHAM.

W. H. Plaister

### MONMOUTHSHIRE.

BLAENAVON.

A. B. Avarne

CALDICOT.

C. Corben

NEWPORT.

W. Basset
C. B. Gratte
H. R. Hudson
O. E. B. Marsh
A. G. Thomas
H. E. Williams

PONTYPOOL.

J. R. Essex
S. B. Mason

RHYMNEY.

T. H. Redwood

RISCA.

G. B. Robathan

TREDEGAR.

G. A. Brown

### NORFOLK.

WALPOLE ST. ANDREWS.

R. Smith

### NOTTINGHAMSHIRE.

NOTTINGHAM.

C. B. Taylor

### OXFORDSHIRE.

OXFORD.

H. P. Symonds

### SOMERSETSHIRE

BATH.

G. A. Bannatyne
C. S. W. Cobbold
T. Cole
F. S. Cowan
S. Craddock
W. M. Ellis
A. E. W. Fox
H. W. Freeman
H. F. A. Goodridge
T. B. Goss
P. King
F. Lace
T. P. Lowe
S. E. Rigg
R. J. H. Scott
W. H. Symons
L. H. Walsh
L. A. Weatherly
J. Wigmore

BLAGDON.

T. O. Halliwell

BRIDGWATER.

F. J. C. Parsons

J. L. K. Pringle

R. H. F. Routh

J. B. Sincock

W. C. Thompson

BRISLINGTON.

B. B. Fox

BURNHAM.

F. C. Berry

CHEDDAR.

E. H. Openshaw
R. W. Statham

CHEW MAGNA.

E. T. Hale

CHILTON POLDEN

G. Campbell

CLEVEDON.

H. J. Capron
T. Davis
W. J. Hill
S. Skinner

CREWKERNE.

E. J. Cave

W. J. Penny

W. W. Webber

CURRY RIVEL.

R. H. Vereker

FRESHFORD.

C. E. S. Flemming

FROME.

A. W. Dalby
J. M. Rattray

ILMINSTER.

C. Munden

KEYNSHAM.

C. Harrison
G. G. D. Willett

### SOMERSETSHIRE *(Continued)*.

LANGPORT.

J. Morgan

LEIGH WOODS.
J. Harvey.

MIDSOMER NORTON.
A. Waugh
A. H. Whicher

MILVERTON.
C. Randolph

NORTH PETHERTON.

C. F. Hawkins

PILL.
T. W. S. Morgan

PORTISHEAD.
W. Monckton
C. A. Wigan

SHEPTON MALLET.
J. T. Hyatt

SOUTH PETHERTON.

S. W. MacIlwaine

TAUNTON.
H. T. S. Aveline
Taunton Hospital
J. A. Macdonald
H. T. Rutherford

TIMSBURY.
F. Woods

WEDMORE.
J. Ford
R. P. Tyley

WELLINGTON.
J. Meredith

WELLS.

W. Fairbanks

W. A. L. Smith

A. L. Wade

WESTON-SUPER-MARE.
H. S. Ballance
C. P. Crouch
G. B. Fraser
E. F. Martin
G. F. Rossiter
R. Roxburgh
J. W. Smith
J. Wallace
R. Wilde

WORLE.
F. St. J. Kemm

WRINGTON.
H. W. Collins

YEOVIL.

P. S. H. Colmer

W. R. M. Semple

### STAFFORDSHIRE.

STAFFORD.
J. Scott

### SUFFOLK.

BECCLES.
F. H. Hudson.

### SURREY.

REDHILL.

C. M. Jessop

FARNHAM.
P. T. Tolputt

VIRGINIA WATER.
S. R. Philipps

### WARWICKSHIRE.

LEAMINGTON.
A. W. W. Hoffman

### WILTSHIRE.

BRADFORD-ON-AVON.
W. J. A. Adye
J. Beddoe

CALNE.
W. S. Batten

DEVIZES.

C. Eddowes

A. M. Gray

FOVANT.
C. Clay

MALMESBURY.
R. Kinneir

MERE.
J. McElfatrick

SWINDON.

W. Howse

J. C. Maclean

WARMINSTER.
R. L. Willcox

### Scotland.

### ARGYLESHIRE.

OBAN.
H. R. McDonald.

### Ireland.

### ANTRIM.

BELFAST.
J. W. Browne
A. Jamison
J. A. Lindsay

### Wales.

### BRECONSHIRE.

TALGARTH.
W. Howells

### CARMARTHENSHIRE.

FERRYSIDE.

P. Williams

LLANELLY.

S. J. Roderick

### CARNARVONSHIRE.

ST. CLEARS.
R. Thomas

### GLAMORGANSHIRE.

ABERDARE.
E. Jones

CARDIFF.
W. T. Edwards
P. R. Griffiths
T. H. Horder
D. R. Paterson
J. A. Phillips
W. Price
A. Rees
A. Sheen
Medical Society
J. L. Thomas
H. R. Vachell
J. Williams

GORSEINON.
T. Mitchell

MAESTEG.
E. Davies
W. H. Thomas

MERTHYR TYDVIL.
W. W. Jones
C. E. G. Simons
J. L. W. Ward

PENARTH.
R. F. Nell

PORTH.
H. N. Davies

SWANSEA.

E. Davies

R. C. Elsworth

E. Le C. Lancaster

TREHARRIS.

W. W. Leigh

### PEMBROKESHIRE.

FISHGUARD.
H. L. Swete

HAVERFORDWEST.
E. P. Phillips

### RADNORSHIRE.

LLANDRINDOD WELLS.
A. G. Greenway

### Belgium.

COURTRAI.
Dr. Lauwers

### Switzerland.

DAVOS-PLATZ.
W. R. Huggard

### Cape Colony.

FORT BEAUFORT.
J. Shaw

### United States.

COLORADO.
S. E. Solly

### Italy.

GENOA.
S. Biamonti

### Egypt.

ALEXANDRIA.
J. Mackie

CAIRO.
M. A. Ruffer

### New Zealand.

LOWER HUTT.
J. R. Purdy

### INDEX.

Abdominal section, Two cases of—Dr. J. R. Thomas, 145.

Abdominale, Du meilleur mode de fermeture de la paroi—E. Doyen (*Review*), 275.

Adenoid vegetations, 339.

Air and its effects upon animal life, The composition of expired—Drs. J. S. Billings, S. W. Mitchell and D. H. Bergey (*Review*), 56.

Alabama, Transactions of the Medical Association of the State of (*Review*), 283.

Alimentary canal, Surgery of the—Dr. A. E. Maylard (*Review*), 179.

Allbutt, Dr. T. C. [Ed.]—A system of medicine (*Reviews*), 56, 264, 344.

Allbutt and W. S. Playfair, Drs. T. C. [Eds.]—A system of gynæcology (*Review*). 351.

Allingham, W. and H. W.—The diagnosis and treatment of diseases of the rectum (*Review*), 182.

Allis, Dr. O. H.—An inquiry into the difficulties encountered in the reduction of dislocations of the hip (*Review*), 66.

Alopecia areata, 254.

American Association of Obstetricians and Gynecologists, Transactions of the (*Review*), 199.

American Dermatological Association, Transactions of the (*Review*), 80.

American Physicians, Transactions of the Association of (*Review*), 283.

American Public Health Association, Reports and papers of the (*Review*), 285.

American Surgical Association, Transactions of the (*Review*) 355.

American year-book of medicine and surgery (*Review*), 200.

Anatomy and landmarks, Surface—Dr. B. C. A. Windle (*Review*), 192

Anderson, Dr. J.—Medical nursing (*Review*). 354,

Angio-neurosis—Dr. W. R. Smith (*Review*), 72.

Annual of the universal medical sciences (*Review*), 557.

Antiseptic principles for nurses—C. E. Richmond (*Review*), 354.

Antitoxic serums, Report of the "Lancet" special commission on the relative strengths of diphtheria (*Review*), 187.

Antre d'Highmore, De la prothèse appliquée au traitement des empyèmes de l'—Dr. S. Dunogier (*Review*), 75.

Antrum, The symptoms and treatment of chronic suppuration of the maxillary—Dr. B. J. Baron, 13.

Aural surgery, Short contributions to—Sir W. B. Dalby (*Review*), 196.

Australasia, The sweating of the medical profession by the friendly societies of—L. Bruck (*Review*), 277.

Autoscopy of the larynx and the trachea—Dr. A. Kirstein (*Review*), 352.

Baby, Our—Mrs. Langton Hewer (*Review*), 195.

Bacteriology, A text-book of—Dr. E. M. Crookshank (*Review*), 192.

Bahadurji, Dr. K. N.—The anomalies and evils of the medical administration of India (*Review*), 75.

Baldness, 254.

Ballantyne, Dr. J. W.—Curettage of the uterus (*Review*), 74.

Balneology and climatology, The journal of (*Review*), 79.

Bandaging and surgical dressing, Elementary—W. Pye (*Review*), 279.

Bannatyne, Dr. G. A.—Rheumatoid arthritis (*Review*), 60.

Barclay, W. M.—Report on surgery, 43.

Baron, Dr. B. J.—Report on laryngology and rhinology, 339; The symptoms and treatment of chronic suppuration of the maxillary antrum, 13.

Barr, Dr. T.—Manual of diseases of the ear (*Review*), 190.

Barrett, Dr. A. W.—Dental surgery (*Review*), 280.
Bath, The Roman Bath at, 379.
Bell, Dr. R.—Sterility (*Review*), 68.
Bergey, Drs. J. S. Billings, S. W. Mitchell and D. H.—The composition of expired air and its effects upon animal life (*Review*), 56.
Billings, S. W. Mitchell and D. H. Bergey, Drs. J.. S.—The composition of expired air and its effects upon animal life (*Review*), 56.
Black, Dr. D. C.—The medical environment (*Review*), 277.
Bocquillon-Limousin, H.—Formulaire des médicaments nouveaux (*Review*), 286.
Boisliniere, Dr. L. C.—Obstetric accidents, emergencies, and operations (*Review*), 187.
Bournemouth in relation to disease, The climate of—Dr. A. Kinsey-Morgan (*Review*), 281.
Bramwell, Dr. B.—Atlas of clinical medicine (*Reieew*), 347.
Bristol health report, 292.
Bristol, Medical Bits of Old, 103, 219, 299, 383.
Bristol Medico-Chirurgical Society, 87, 206, 362.
British Balneological and Climatological Society, Transactions of the (*Review*), 79.
British Medical Benevolent Fund, Annual report of the (*Review*), 360.
British Orthopædic Society, Transactions of the (*Review*), 356.
Brockbank, Dr. E. M.—On gall-stones or cholelithiasis (*Review*), 179.
Bruck, L.—The sweating of the medical profession by the friendly societies of Australasia (*Review*), 277.
Buck, Dr. A. H.—A vest-pocket medical dictionary (*Review*), 278.
Buret, Dr. F.—Syphilis in the middle ages and in modern times (*Review*), 53.
Butler, Dr. G. F.—A text-book of materia medica, therapeutics, and pharmacology (*Review*), 352.
Butler-Smythe, A. C.—A first series of fifty-four consecutive ovariotomies, with fifty-three recoveries (*Review*), 279.

Calculi, The effects of the Röntgen rays on—Dr. J. Swain, 1.
Cancer, Operations for—W. W. Cheyne (*Review*), 63.
Carwardine, T.—The morphology and pathology of the pharyngeal pouch of Rathke, 310.
Chemical notes and equations—G. H. Gemmell (*Review*), 70.
Chemistry for the Conjoint Board, Guide to the examination in practical—P. A. E. Richards (*Review*), 194.
Chemistry, The progress of medical—Dr. J. L. W. Thudichum (*Review*), 278.
Chest, The surgery of the—S. Paget (*Review*), 65.
Cheyne, W. W.—The objects and limits of operations for cancer (*Review*), 63; The treatment of wounds, ulcers, and abscesses (*Review*), 279.
Chloroform, The "Lancet" and the Hyderabad commissions on (*Review*), 184.
Cincinnati hospital, Annual report of the (*Review*), 78.
Clarke, Dr. J. M.—A case of acute miliary tuberculosis, 148; Reports on medicine, 38, 246.
Clarkson, Dr. A.—A text-book of histology, descriptive and practical (*Review*), 55.
Clinical excerpts (*Review*), 203.
Clinical Society of London, Transactions of the (*Review*), 199.
Cliniques, Royal Infirmary—Dr. A. James (*Review*), 194.
Club-foot, Paralytic, 161.
Clubs, The battle of the (*Review*), 176.
Colliery explosion, The causes of death in five cases in a—Dr. T. Mitchell, 235.
Colman, Dr. W. S.—Section cutting and staining (*Review*), 193.
Compressed air illness—Dr. E. H. Snell (*Review*), 61.
Consumptives, Sanatoria for—A. von Jaruntowsky (*Review*), 281.
Corfield, Dr. W. H.—Disease and defective house sanitation (*Review*), 76.
Cornwall as a winter resort—E. Kitto [Ed.] (*Review*), 282.
Crookshank, Dr. E. M.—A text-book of bacteriology (*Review*), 192.
Cross, F. R.—Report on ophthalmology, 48.
Current from the main—Dr. W. S. Hedley (*Review*), 72.
Cycling as a cause of heart disease—Dr. G. Herschell (*Review*), 279.

Dalby, Sir. W. B.—Short contributions to aural surgery (*Review*), 196.
Dalton, Dr. N.—Aids to medicine (*Review*), 71.
Deafness, giddiness, and noises in the head—Dr. E. and C. Woakes (*Review*), 276.
Deformities—A. H. Tubby (*Review*), 271.
Delanglade, Dr. É.—De la luxation congénitale du fémur (*Review*), 272.
Dental surgery—Dr. A. W. Barrett (*Review*), 280.
Dermatological Society of Great Britain and Ireland, Transactions of the (*Review*), 200.
Dermatology, Report on—Dr. H. Waldo, 254.
Devon and Exeter Medico-Chirurgical Society, 93.
Diagnosis, Clinical—Dr. W. G. A. Robertson (*Review*), 71.
Dictionary, A vest-pocket medical—Dr. A. H. Buck (*Review*), 278.
Dictionary, The student's medical—Dr. G. M. Gould (*Review*), 260.
Dorland, Dr. W. A. N.—A manual of obstetrics (*Review*), 273.
Doyen, E.—Traitement des suppurations pelviennes. De l'hystérectomie abdominale totale. Traitement chirurgical des rétrodéviations utérines. Du meilleur mode de fermeture de la paroi abdominale (*Review*), 275.
Doyne, R. W.—Notes on the more common diseases of the eye (*Review*), 195.
Dress and health—C. M. Jessop (*Review*), 76.
Dunogier, Dr. S.—De la prothèse appliquée au traitement des empyèmes de l'antre d'Highmore (*Review*), 75.

Ear, Chronic catarrh of the middle, 342.
Ear, Diseases of the—Dr. T. Barr (*Review*), 190; Dr. U. Pritchard (*Review*), 74.
Ear, Injuries and diseases of the—M. Yearsley (*Review*), 358.
Eccles, W. McA. — Elementary anatomy and surgery for nurses (*Review*), 70.
Edinburgh hospital reports (*Review*), 78.
Edinburgh medical journal (*Review*), 285.
Edinburgh Obstetrical Society, Transactions of the (*Review*), 357
Embolism of the central artery of the retina, 48.
Epidemiological Society of London, Transactions of the (*Review*), 285.
Eshner, Drs. J. C. Wilson and A. A. [Eds.]—An American text-book of applied therapeutics (*Review*), 183.
Eucaine hydrochlorate in ophthalmic practice, 52.
Ewart, Dr. W.—Gout and goutiness (*Review*), 177.
Experimental medicine, Journal of (*Review*), 202.
Eye, Diseases of the—R. W. Doyne (*Review*), 195.

Fémur, De la luxation congénitale du—Dr. É. Delanglade (*Review*), 272.
Fernie, Dr. W. T.—Herbal simples (*Review*), 280.
Firth, Dr. J. L.—Paralysis of the respiratory centre from intracranial pressure, 139.
Fisher, Dr. T.—Report on medicine, 33.
Fitz, Drs. H. C. Wood and R. H.—The practice of medicine (*Review*), 262.
Food in health and disease—Dr. I. B. Yeo (*Review*), 76.
Formalin, Treatment of purulent ophthalmia by, 52.
Fothergill, Dr. W. E.—Manual of midwifery (*Review*), 67.
Foxwell, Dr. A.—The enlarged cirrhotic liver (*Review*), 194.
Freyer, Dr. P. J. — The modern treatment of stone in the bladder by litholapaxy (*Review*), 73.
Frontal bone, Compound depressed fracture of—A. W. Prichard, 27.
Fyffe, Dr. W. J.—Fifteen years' experience of infectious diseases in a public school, 221.

Gärtner, Dr. A. — Leitfaden der hygiene (*Review*), 69.
Gall-stones—Dr. E. M. Brockbank (*Review*), 179.
Gant, Dr. S. G.—Diseases of the rectum (*Review*), 181.
Gemmell, G. H.—Chemical notes and equations (*Review*), 70.
Glandular fever, 33.
Goodall and J. W. Washbourn, Drs. E. W.—A manual of infectious diseases (*Review*), 191.

Gould, Dr. G. M.—The student's medical dictionary (*Review*), 260.
Gout and goutiness—Dr. W. Ewart (*Review*), 177.
Grant College Medical Society, Transactions of the (*Review*), 79.
Guy's hospital reports (*Review*), 282.
Gynæcology, A system of—Drs. T. C. Allbutt and W. S. Playfair [Eds.] (*Review*), 351.
Gynæcology and obstetrics, Report on—Dr. W. C. Swayne, 168.
Gynecology, Practical and operative —Dr. J. C. Webster (*Review*), 190.

Hæmophilia with joint-lesions, A case of—Dr. J. E. Shaw, 240.
Haig-Brown, Florence A.—Hints on elementary physiology (*Review*), 193.
Halliburton, Dr. W. D.—Kirkes' hand-book of physiology (*Review*), 54.
Harsant, W. H.—Report on otology, 342; Report on surgery, 251.
Heart disease, Cycling as a cause of—Dr. G. Herschell (*Review*), 279.
Hedley, Dr. W. S.—Current from the main (*Review*), 72; The hydro-electric methods in medicine (*Review*), 72.
Hepatic and intestinal surgery, Cases of—C. A. Morton, 317.
Herbal simples—Dr. W. T. Fernie (*Review*), 280.
Herschell, Dr. G.—Cycling as a cause of heart disease (*Review*), 279.
Hewer, Mrs. Langton—Our baby (*Review*), 195.
Hip, Dislocations of the—Dr. O. H. Allis (*Review*), 66.
Histology, A text-book of—Dr. A. Clarkson (*Review*), 55.
Hospital annual, Burdett's (*Review*), 286.
Howell, Dr. W. H. [Ed.] — An American text-book of physiology (*Review*), 261.
Hughes, Amy—Practical hints on district nursing (*Review*), 358.
Hyde, Dr. S.—Rheumatoid arthritis (*Review*), 194.
Hyderabad commissions on chloroform, The "Lancet" and the (*Review*), 184.
Hydro-electric methods in medicine —Dr. W. S. Hedley (*Review*), 72.
Hygiene, Leitfaden der — Dr. A. Gärtner (*Review*), 69.
Hystérectomie abdominale totale, De l'—E. Doyen (*Review*), 275.
Hysterectomy for malignant disease, Vaginal—Dr. W. H. C. Newnham, 24.
Hysterical disease of both hips and knees—C. A. Morton, 30.

India, The anomalies and evils of the medical administration of—Dr. K. N. Bahadurji (*Review*), 75.
India, The consumption of opium in (*Review*), 196.
Infectious Disease (Notification) Act, Difficulties under the (*Review*), 197.
Infectious diseases, A manual of—Drs. E. W. Goodall and J. W. Washbourn (*Review*), 191.
Infectious diseases in a public school —Dr. W. J. Fyffe, 221.
Intestinal surgery, Cases of hepatic and—C. A. Morton, 317.
Intubation in adults, Two cases of—H. F. Mole, 237.
Iowa State Medical Society, Transactions of the (*Review*)), 355.

Jackson, Dr. E.—Skiascopy and its practical application to the study of refraction (*Review*), 275.
Jacobson, W. H. A.—The operations of surgery (*Review*), 269.
James, Dr. A. — Royal Infirmary cliniques (*Review*), 194.
Jaruntowsky, A. von—The private sanatoria for consumptives (*Review*), 281.
Jenner, 70.
Jessop, C. M.—Dress and health (*Review*), 76.
Johns Hopkins hospital reports (*Review*), 77.
Johnson, Sir G.—The pathology of the contracted granular kidney (*Review*), 60.
Johnson, Theodora—The Swedish system of physical education (*Review*), 358.

Kidney, Cirrhosis of the, 155.
Kidney disease, Tubercular, 160.
Kidney, The pathology of the contracted granular—Sir G. Johnson (*Review*), 60.
Kinsey-Morgan, Dr. A.—The climate of Bournemouth in relation to disease (*Review*), 281.
Kirkes' hand-book of physiology—Dr. W. D. Halliburton (*Review*), 54.

Kirstein, Dr. A.—Autoscopy of the larynx and the trachea (*Review*), 352.
Kitto, E. [Ed.]—Cornwall as a winter resort (*Review*), 282.
Knauer, Dr. O.—Über puerperale psychosen (*Review*), 350.

Laryngeal growths, 341.
Laryngology and rhinology, Report on—Dr. B. J. Baron, 337.
Larynx and the trachea, Autoscopy of the—Dr. A. Kirstein (*Review*), 352.
Lee, A. B.—The microtomist's vade-mecum (*Review*), 262.
Lersch, Dr. B. M.—Geschichte der volksseuchen (*Review*), 53.
Library of the Bristol Medico-Chirurgical Society, 96, 214, 289, 367.
Ligaments—J. B. Sutton (*Review*), 344.
Litholapaxy, The modern treatment of stone in the bladder by—Dr. P. J. Freyer (*Review*), 73.
Liver, Cirrhosis of the, 154.
Liver, gall-bladder, and biliary system, Diseases of the—H. J. Waring (*Review*), 349.
Liver, The enlarged cirrhotic—Dr. A. Foxwell (*Review*), 194.
Local medical notes, 99, 217, 292, 379.
London, Transactions of the Clinical Society of (*Review*), 199.
London, Transactions of the Epidemiological Society of (*Review*), 285.
London, Transactions of the Medical Society of (*Review*), 284.
London, Transactions of the Obstetrical Society of (*Review*), 79.
Lunacy practice, Some points in—Dr. J. E. Shaw, 301.

Maclagan, Dr. T. J.—Rheumatism (*Review*), 59.
Mahlendorff, P.—Voice production (*Review*), 196.
Materia medica, therapeutics, and pharmacology, A text-book of—Dr. G. F. Butler (*Review*), 352.
Matron's course, The—Miss S. E. Orme (*Review*), 354.
Maylard, Dr. A. E.—The surgery of the alimentary canal (*Review*), 179.
Medical annual (*Review*), 81.
Medical environment, The—Dr. D. C. Black (*Review*), 277.
Médicaments nouveaux, Formulaire des — H. Bocquillon - Limousin (*Review*), 286.
Medicine, A system of—Dr. T. C. Allbutt [Ed.] (*Reviews*), 56, 264, 344.
Medicine, Aids to—Dr. N. Dalton (*Review*), 71.
Medicine, Atlas of clinical—Dr. B. Bramwell (*Review*), 347.
Medicine, Reports on—Dr. J. M. Clarke, 38, 246; Dr. T. Fisher, 33; Dr. G. Parker, 154; Dr. R. S. Smith, 326.
Medicine, The practice of—Drs. H. C. Wood and R. H. Fitz (*Review*), 262.
Medicine, The profession of—Dr. C. West (*Review*), 175.
Menopause and its disorders, The—Dr. A. D. L. Napier (*Review*), 274.
Metatarsalgia, 251.
Michigan State Medical Society, Transactions of the (*Review*), 198.
Microtomist's vade-mecum, The—A. B. Lee (*Review*), 262.
Middlesex hospital journal (*Review*), 82.
Midwifery, Manual of—Dr. W. E. Fothergill (*Review*), 67.
Mitchell and D. H. Bergey, Drs. J. S. Billing, S. W.—The composition of expired air and its effects upon animal life (*Review*), 56.
Mitchell, Dr. T.—The causes of death in five cases in a colliery explosion, 235.
Mole, H. F.—Two cases of intubation in adults, 237.
Morton, C. A.—A case of hysterical disease of both hips and both knees, with extreme distortion of the lower limbs, 30; Cases of hepatic and intestinal surgery, 317; Report on surgery, 161.
Morton's painful foot, 251.
Moure, Dr. E. J.—Du traitement chirurgical de la surdité et des bourdonnements (*Review*), 75.
Moyes, Dr. J.—Medicine and kindred arts in the plays of Shakespeare (*Review*), 353.
Murray, Dr. W.—Rough notes on remedies (*Review*), 280.
Murrell, Dr. W.—What to do in cases of poisoning (*Review*), 280.

Nall, Dr. S. — Aids to obstetrics (*Review*), 73.

Napier, Dr. A. D. L.—The menopause and its disorders (*Review*), 274.
New Hampshire Medical Society, Transactions of the (*Review*), 283.
Newnham, Dr. W. H. C.—A case of ruptured tubal pregnancy, 152; Three cases of vaginal hysterectomy for malignant disease, 24.
New York, Transactions of the Medical Society of the State of (*Review*), 197.
Nurses, Antiseptic principles for—C. E. Richmond (*Review*), 354.
Nurses' diary (*Review*), 82.
Nursing, District — Amy Hughes (*Review*), 358.
Nurses, Elementary anatomy and surgery for—W. McA. Eccles (*Review*), 70.
Nursing, Medical—Dr. J. Anderson (*Review*), 354.
Nursing, Practical points in—Emily M. A. Stoney (*Review*), 276.

Obstetric accidents, emergencies, and operations—Dr. L. C. Boisliniere (*Review*), 187.
Obstetrical Society of London, Transactions of the (*Review*), 79.
Obstetrics, A manual of—Dr. W. A. N. Dorland (*Review*), 273.
Obstetrics, Aids to — Dr. S. Nall (*Review*), 73.
Obstetrics, Report on gynæcology and—Dr. W. C. Swayne, 168.
Ohio State Medical Society, Transactions of the (*Review*), 359.
Ophthalmological Society of the United Kingdom, Transactions of the (*Review*), 356.
Ophthalmia, Treatment by formalin of purulent, 52.
Ophthalmology, Report on—F. R. Cross, 48.
Opium in India, The consumption of (*Review*), 196.
Orme, Miss S. E.—The matron's course (*Review*), 354.
Otology, Report on—W. H. Harsant, 342.
Ovariotomies, A first series of fifty-four consecutive—A. C. Butler-Smythe (*Review*), 279.

Paget, S.—The surgery of the chest (*Review*), 65.
Pancreatitis, Acute, 43.
Parker, Dr. G.—Report on medicine, 154.
Pelviennes, Traitement des suppurations—E. Doyen (*Review*), 275.
Peritonitis, 334.
Phelps's operation, 164.
Philadelphia, Transactions of the College of Physicians of (*Review*), 78.
Physical education, The Swedish system of—Theodora Johnson (*Review*), 358.
Physiology, An American text-book of—Dr. W. H. Howell [Ed.] (*Review*), 261.
Physiology, Hints on elementary—Florence A. Haig-Brown (*Review*), 193.
Physiology, Kirkes' hand-book of—Dr. W. D. Halliburton (*Review*), 54.
Pituitary extract in acromegaly, 251.
Playfair, Drs. T. C. Allbutt and W. S. [Eds.] — A system of gynæcology (*Review*), 351.
Plumbers' work, Report of the "Lancet" special commission on (*Review*), 197.
Plymouth Medical Society, 94, 366.
Poisoning, What to do in cases of—Dr. W. Murrell (*Review*), 280.
Pregnancy, Ruptured tubal — Dr. W. H. C. Newnham, 152.
Prichard, A. W.—Two cases of compound depressed fracture of frontal bone, 27.
Pritchard, Dr. U.—Diseases of the ear (*Review*), 74.
Puerperale psychosen, Über—Dr. O. Knauer (*Review*), 350.
Pye, W. — Elementary bandaging and surgical dressing (*Review*), 279.

Rathke, The pharyngeal pouch of—T. Carwardine, 310.
Rectum, Diseases of the—W. and H. W. Allingham (*Review*), 182; Dr. S. G. Gant (*Review*), 181.
Refraction, Skiascopy and its practical application to the study of—Dr. E. Jackson (*Review*), 275.
Remedies, Rough notes on—Dr. W. Murray (*Review*), 280.
Renal and urinary diseases, Lectures on—Dr. R. Saundby (*Review*), 266.
Respiratory centre from intracranial pressure, Paralysis of the—Dr. J. L. Firth, 139.
Retina, Embolism of the central artery of the, 48.
Revue philanthropique, La (*Review*), 203.

Rheumatism—Dr. T. J. Maclagan (*Review*), 59.
Rheumatoid arthritis, 327; Dr. G. A. Bannatyne (*Review*), 60; Dr. S. Hyde (*Review*), 194.
Rhinitis, Atrophic, 340.
Rhinology, Report on laryngology and—Dr. B. J. Baron, 339.
Richards, P. A. E.—Guide to the examination in practical chemistry for the Conjoint Board (*Review*), 194.
Richmond, C. E.—Antiseptic principles for nurses (*Review*), 354.
Robertson, Dr. W. G. A.—Clinical diagnosis (*Review*), 71.
Roentgen ray, Archives of the (*Review*), 359.
Röntgen rays on calculi, The effects of the—Dr. J. Swain, 1.

Sanitation, Disease and defective house—Dr. W. H. Corfield (*Review*), 76.
Saundby, Dr. R.—Lectures on renal and urinary diseases (*Review*), 266.
Scarlet fever, Secondary rashes in—Dr. J. O. Symes, 20.
School, Infectious diseases in a public—Dr. W. J. Fyffe, 221.
Scopolamine on the iris and ciliary muscle, Action of, 51.
Scottish medical and surgical journal (*Review*), 82.
Scraps, 101, 218, 295, 383.
Seborrhœa, 259.
Section cutting and staining—Dr. W. S. Colman (*Review*), 193.
Senn, N.—The pathology and surgical treatment of tumors (*Review*), 270.
Serumtherapy, 38.
Shakespeare, Medicine in—Dr. J. Moyes (*Review*), 353.
Shaw, Dr. J. E.—A case of hæmophilia with joint-lesions, 240; Some points in lunacy practice in relation to the general practitioner, 301.
Sick, Preparations for the, 83, 203, 286, 361.
Skiascopy and its practical application to the study of refraction—Dr. E. Jackson (*Review*), 275.
Smith, James Greig (In Memoriam), 105; Memorial to, 295.
Smith, Dr. R. S.—Report on medicine, 326.
Smith, Dr. W. R.—Angio-neurosis: being studies in diseases of the vaso-motor system (*Review*), 72.
Snell, Dr. E. H.—Compressed air illness, or so-called caisson disease (*Review*), 61.
Societies: Bristol Medico-Chirurgical, 87, 206, 362; Devon and Exeter Medico-Chirurgical, 93; Plymouth Medical, 94, 366.
South Carolina Medical Association, Transactions of the (*Review*), 284.
Spas, watering places, and health resorts, Dr. Tiebel's official handbook to (*Review*), 77.
Splenic extract in exophthalmic goître, 251.
Sterility—Dr. R. Bell (*Review*), 68.
Stomach, Dilatation of the, 158.
Stoney, Emily A. M. — Practical points in nursing, for nurses in private practice (*Review*), 276.
St. Thomas's hospital reports (*Review*), 354.
Suprarenal extract in medicine, 249.
Surdité et des bourdonnements, Du traitement chirurgical de la—Dr. E. J. Moure (*Review*), 75.
Surgery, Reports on—W. M. Barclay, 43; W. H. Harsant, 251; C. A. Morton, 161; Dr. J. Swain, 334.
Surgery, The operations of—W. H. A. Jacobson (*Review*), 269.
Surgical emergencies — P. Swain (*Review*), 63.
Sutton, J. B.—Ligaments (*Review*), 344.
Swain, Dr. J.—Report on surgery, 334; The effects of the Röntgen rays on calculi, 1.
Swain, P. — Surgical emergencies (*Review*), 63.
Swayne, Dr. W. C. — Report on gynæcology and obstetrics, 168.
Swedish system of physical education, The — Theodora Johnson (*Review*), 358.
Symes, Dr. J. O.—Secondary rashes in scarlet fever, 20.
Syphilis in the middle ages and in modern times—Dr. F. Buret (*Review*), 53.

Tachycardia, Paroxysmal — Dr. P. W. Williams, 122.
Talipes equino-varus, Congenital, 162.
Tarsectomy, 167.
Therapeutics, An American textbook of applied—Drs. J. C. Wilson and A. A. Eshner [Eds.] (*Review*), 183.
Thomas, Dr. J. R. — Two cases of abdominal section, 145.

Thudichum, Dr. J. L. W.—The progress of medical chemistry (*Review*), 278.
Thymus extract in medicine, 250.
Thyroid extract in medicine, 246.
Treatment (*Review*), 203.
Tubby, A. H. — Deformities: a treatise on orthopædic surgery (*Review*), 271.
Tuberculosis, Acute miliary — Dr. J. M. Clarke, 148.
Tuberculosis, 329.
Tumors, The pathology and surgical treatment of—N. Senn (*Review*), 270.
Turbinotomy, 341.

Uræmia, 159.
Utérines, Traitement chirurgical des rétrodéviations — E. Doyen (*Review*), 275.
Uterus, Curettage of the—Dr. J. W. Ballantyne (*Review*), 74.
Uterus, Displacements of the, 168.

Ventrofixation of the uterus, 168.
Voice production—P. Mahlendorff (*Review*), 196.
Volksseuchen, Geschichte der—Dr. B. M. Lersch (*Review*), 53.

Waldo, Dr. H.—Report on dermatology, 254.
Waring, H. J.—Diseases of the liver, gall-bladder, and biliary system (*Review*), 349.
Washbourn, Drs. E. W. Goodall and J. W.—A manual of infectious diseases (*Review*), 191.
Webster, Dr. J. C. — Practical and operative gynecology (*Review*), 190.
West, Dr. C. — The profession of medicine (*Review*), 175.
West London Medico-Chirurgical Society, Proceedings of the (*Review*), 199.
Williams, Dr. P. W.—Paroxysmal tachycardia, 122.
Wilson and A. A. Eshner, Drs. J. C. [Eds.] — An American text-book of applied therapeutics (*Review*), 183.
Windle, Dr. B. C. A. — Surface anatomy and landmarks (*Review*), 192.
Woakes, Dr. E. and C.—Deafness, giddiness, and noises in the head (*Review*), 276.
Wood and R. H. Fitz, Drs. H. C.—The practice of medicine (*Review*), 262.
Wounds, ulcers, and abscesses, The treatment of—W. W. Cheyne (*Review*), 279.

Year-book of scientific societies (*Review*), 286.
Year-book of treatment (*Review*), 81.
Yearsley, M.—Injuries and diseases of the ear (*Review*), 358.
Yeo, Dr. I. B.—Food in health and disease (*Review*), 76.

J. W. Arrowsmith, Printer, Quay Street, Bristol.

### *PUBLICATIONS RECEIVED.*

From J. W. Arrowsmith, Bristol:

*Abdominal Surgery.* By J. Greig Smith, M.A., F.R.S.E. Sixth Edition. Edited by James Swain, M.S., M.D. Lond. Two volumes. 1897.

From Baillière, Tindall and Cox, London:

*Perineal Operations.* By W. J. Stewart McKay, M.B., M.Ch., B.Sc. 1897.
*The Oxygen Treatment for Wounds, Ulcers, Burns, Scalds, Lupus, and Diseases of the Nose, Eye, and Ear.* By George Stoker. 1897.
*Heart Disease.* By Sir William H. Broadbent, Bart., M.D., F.R.S., and John F. H. Broadbent, M.A., M.D. 1897.
*The Practice of Massage.* By A. Symons Eccles, M.B. Second Edition. 1898 [*sic*].
*Air, Food, and Exercises.* By A. Rabagliati, M.A., M.D. [n.d.]

From John Bale, Sons & Danielsson, Ltd., London:

*The Journal of Balneology and Climatology.* October, 1897.

From The Bath Corporation, Bath:

*Bath as a Health Resort.* [n.d.]

From Friedr. Bayer & Co., Elberfeld:

*Clinical Excerpts.* September, October, 1897.

From William Blackwood and Sons, Edinburgh:

*An Indian Romance: A Lesson of the Famine.* 1897. [Reprint.]

From Burgoyne, Burbidges & Co., London:

*Xeroform.* 1897.
*Guaiacol Carbonate and Creosote Carbonate.* 1897.

From California Medical College, California:

*California Medical Journal.* October, November, 1897.

From Cassell and Co., Limited, London:

*Clinical Methods.* By Robert Hutchison, M.D., and Harry Rainy, M.A., F.R.S.E. 1897.
*The South African Climate.* By William C. Scholtz, M.D. 1897.

From J. & A. Churchill, London:

*Practice of Midwifery.* By Haydn Brown. 1897.
*Theory and Practice of Surgery.* By William John Walsham, M.B., C.M. Sixth Edition. 1897.
*Human Nature: its Principles and the Principles of Physiognomy.* By Physicist. Part I. 1897.

From Church Missionary Society, London:

*Mercy and Truth.* October—December, 1897.

From William F. Clay, Edinburgh:

*Reports from the Laboratory of the Royal College of Physicians of Edinburgh.* Vols. V., 1894, VI., 1897.
*Medical Diagnosis.* By J. J. Graham Brown, M.D. Fourth Edition. 1897.

From The F. A. Davis Company, Philadelphia:

*An Epitome of the History of Medicine.* By Roswell Park, A.M., M.D. 1897.

From The Detroit Journal Office, Detroit:

*The Detroit Journal.* August 17, 1897.

From Eduardo Dublan, Mexico:

*Boletin del Consejo superior de Salubridad.* Tomo II. Núm. 11, 12. III. 2, 4. 1897.

From Paul Dupont, Paris:

*Sur les Applications nouvelles du Courant ondulatoire en Thérapeutique générale.* Par le Dr. G. Apostoli. 1897.

*Essai de Synthèse électrothérapique de la Franklinisation et des Courants de Haute fréquence.* Par le Dr. G. Apostoli. 1897.

*Note sur trois Cas intéressants de Nœvus traités et guéris par la Galvanopuncture.* Par le Dr. G. Apostoli. 1897.

From Dr. L. Webster Fox, Philadelphia:

*Epiphora, or Watery Eye; Lachrymal Abscess; Necrosis of the Bony Walls of the Lachrymal Canal; Implantation of a Glass Ball for the Support of an Artificial Eye; Grattage for the Radical Cure of Granular Lids.* By L. Webster Fox, M.D. 1897. [Reprint.]

From G. Gounouilhou, Bordeaux:

*Sur trois Cas de Complications intra-craniennes d'Origine otique.* Par le Dr. E. J. Moure. 1897.

From Charles Griffin and Company, Limited, London:

*Outlines of the Diseases of Women.* By John Phillips, M.A., M.D. Second Edition. 1897.

From Dr. John Homans, Boston, U.S.A.:

*Recurrent Gall-Stones. Angioma of Spleen. Excision of Cæcum.* By John Homans, M.D. [n.d.]

From Ingram Brothers, London:

*The English Illustrated Magazine.* October—December, 1897.

From Joseph Jacobs, Atlanta:

*A Distinguished Physician-Pharmacist—His Great Discovery, Ether-Anæsthesia.* By Joseph Jacobs. 1897.

From Judd & Detweiler, Washington:

*Scopelism.* By Robert Fletcher, M.D. 1897. [Reprint.]

From Henry Kimpton, London:

*Practical Diagnosis.* Second Edition. By Hobart Amory Hare, M.D., B.Sc. 1897.
*Text-Book of Practical Therapeutics.* By Hobart Amory Hare, M.D., B.Sc. Sixth Edition. 1897.
*A System of Practical Medicine.* Edited by Alfred Lee Loomis, M.D., LL.D., and William Gilman Thompson, M.D. Vols. I., II. 1897.

From Knoll & Co., Ludwigshafen, a/Rh.:

*Zur Wirkung des Diuretins.* Von Dr. M. Steiner. 1897. [Reprint.]

*Neuere Mittheilungen über einige Organ-Präparate.* I. Thyradēn. II. Ovaradēn.

From Alois Kremel, Vienna:

*Die Dieterich-'schen Eisenmanganpräparate heutiger Therapie.* Von Prof. G. Scognamiglio. 1897. [Reprint.]

From Dr. J. J. Lawrence, St. Louis:

*The Medical Brief.* November, 1897.

From H. K. Lewis, London:

*Ringworm and Alopecia Areata.* By H. Aldersmith, M.B. Fourth Edition. 1897.
*A Manual of Obstetric Practice.* By Professor A. Dührssen, M.D. Translated and Edited from the Sixth Edition by John W. Taylor and Frederick Edge, M.D. 1897.
*Hygiene and Public Health.* By Louis C. Parkes, M.D., D.P.H. Fifth Edition. 1897.
*The Sanitary Inspector's Handbook.* By Albert Taylor. Second Edition. 1897.
*Handbook of Therapeutics.* By Sydney Ringer, M.D., F.R.S., and Harrington Sainsbury, M.D. Thirteenth Edition. 1897.
*Mastoid Abscesses and their Treatment.* By A. Broca, M.D., and F. Lubet-Barbon, M.D. Translated and Edited by Henry J. Curtis, B.S., M.D. 1897.

From E. Licéaga, Mexico:

*Memoria de los Trabajos ejecutados por el Consejo Superior de Salubridad en el Año de* 1895. 1897.

From J. B. Lippincott Company, Philadelphia:

*The Practice of Surgery.* By Henry R. Wharton, M.D., and B. Farquhar Curtis, M.D. 1898 [*sic*].
*System of Diseases of the Eye.* Edited by William F. Norris, A.M., M.D., and Charles A. Oliver, A.M., M.D. Vols. I., II. 1897.

From Longmans, Green, & Co., London:

*Wounds in War.* By Surgeon-Colonel W. F. Stevenson, A.B., M.B., B.Ch. 1897.
*A Handbook of Midwifery.* By W. R. Dakin, M.D., B.S. 1897.

From Robert E. Lynch, Philadelphia:

*Report of the Kensington Hospital for Women,* 1895–96.

From Macmillan and Co., Limited, London:

*A System of Medicine.* Edited by Thomas Clifford Allbutt, M.A., M.D., LL.D., F.R.S. Volume IV. 1897.
*The Practitioner's Handbook of Treatment.* By the late J. Milner Fothergill, M.D. Fourth Edition. Edited by William Murrell, M.D. 1897.

From Marriott & Williams, London:

*The Science and Art of Adjustment between the Producing and Reflecting Vocal Apparatus.* By Paul Mahlendorff. 1897.

From "The Medical Times," Ltd., London:

*The Medical Times and Hospital Gazette.* December 4, 1897.

### PUBLICATIONS RECEIVED *(continued)*.

From The Medico-Hygienic Inventions Company, Limited, London:

*Aerial Disinfection.* [n.d.]

From Peter Möller, London:

*A New Era in Cod Liver Oil Manufacture.* [n.d.]

From Oficina Tip. de la Secretaria de Fomento, Mexico:

*Revista Médica.* Tomo X. Núm. 5—9. 1897.

From Dr. Phocas, Lille:

*Considérations sur le fonctionnement de l'Hôpital de campagne envoyé de Paris à la guerre gréco-turque.* M. le docteur Phocas. 1897.

From La Presse Scientifique Internationale, Paris:

*Des relations de l'appendicite et des affections des annexes.* Par M. le Dr. H. Delagénière. 1897. [Compte rendu.]
*De l' hystérectomie abdominale totale comme temps préliminaire de l'extirpation des kystes papillaires intraligamentaires.* Par M. le Dr. H. Delagénière. 1897. [Compte rendu.]
*Des lithiases biliaires latentes et de leur traitement chirurgical.* Par M. le Dr. H. Duret. 1897. [Compte rendu.]

From The Rebman Publishing Company, Limited, London:

*Archives of the Roentgen Ray.* November, 1897.
*Radiography in Marine Zoology. The British Echinodermata.* By R. Norris Wolfenden, M.D. 1897.
*Cardiac Failure.* By Alexander Morison, M.D. 1897.
*Some Points in the Anatomy, Pathology, and Surgery of Intussusception.* By D'Arcy Power, M.A., M.B. 1898 [*sic*].

From J. M. Richards, London:

*Medical Reprints.* September—November, 1897.

From John Ritchie & Co., Edinburgh:

*The Scotsman.* October 5, 1897.

From The Scientific Press, Limited, London:

*Fever Nursing.* By William Harding, M.D. 1897.
*Hygiene for Students and Nurses.* By John Glaister, M.D., D.P.H. 1897.
*Burdett's Official Nursing Directory*, 1898.

From Smith, Elder & Co., London:

*Spinal Caries.* By Noble Smith. Second Edition. 1897.
*Nervous Affections of the Hand, and other Studies.* By George Vivian Poore, M.D. 1897.

From Steenske Bogtrykkeri, Christiania:

*Studier over Gummos ("Tertiær") Syfilis.* Af Kristian Gron. 1897.

From Stories, Limited, London:

*Stories.* Vol. I. No. 1. November 6, 1897.

From T. Fisher Unwin, London:

*John Hunter.* By Stephen Paget. 1897.

From Leopold Voss, Hamburg:

*Uber weitere Erfahrungen mit Ichthalbin (Ichthyoleiweiss).* Von Dr. Arnold Sack. 1897. [Reprint.]

From Western Gazette Publishing Company, Denver:

*Western Medical and Surgical Gazette.* Vol. I. No. 1. November, 1897.

